# The BRCT Domain of PARP-1 Is Required for Immunoglobulin Gene Conversion

**DOI:** 10.1371/journal.pbio.1000428

**Published:** 2010-07-20

**Authors:** Marcia N. Paddock, Ben D. Buelow, Shunichi Takeda, Andrew M. Scharenberg

**Affiliations:** 1 Department of Immunology, University of Washington, Seattle, Washington, United States of America; 2 Center for Immunity and Immunotherapies, Seattle Children's Hospital Research Institute, Seattle, Washington, United States of America; 3 Crest Laboratory, Department of Radiation Genetics, Faculty of Medicine, Kyoto University, Sakyo-ku, Kyoto, Japan; Scripps Research Institute, United States of America

## Abstract

Genetic variation at immunoglobulin (*Ig*) gene variable regions in B-cells is created through a multi-step process involving deamination of cytosine bases by activation-induced cytidine deaminase (AID) and their subsequent mutagenic repair. To protect the genome from dangerous, potentially oncogenic effects of off-target mutations, both AID activity and mutagenic repair are targeted specifically to the *Ig* genes. However, the mechanisms of targeting are unknown and recent data have highlighted the role of regulating mutagenic repair to limit the accumulation of somatic mutations resulting from the more widely distributed AID-induced lesions to the *Ig* genes. Here we investigated the role of the DNA damage sensor poly-(ADPribose)-polymerase-1 (PARP-1) in the repair of AID-induced DNA lesions. We show through sequencing of the diversifying *Ig* genes in PARP-1^−/−^ DT40 B-cells that PARP-1 deficiency results in a marked reduction in gene conversion events and enhanced high-fidelity repair of AID-induced lesions at both *Ig* heavy and light chains. To further characterize the role of PARP-1 in the mutagenic repair of AID-induced lesions, we performed functional analyses comparing the role of engineered PARP-1 variants in high-fidelity repair of DNA damage induced by methyl methane sulfonate (MMS) and the mutagenic repair of lesions at the *Ig* genes induced by AID. This revealed a requirement for the previously uncharacterized BRCT domain of PARP-1 to reconstitute both gene conversion and a normal rate of somatic mutation at *Ig* genes, while being dispensable for the high-fidelity base excision repair. From these data we conclude that the BRCT domain of PARP-1 is required to initiate a significant proportion of the mutagenic repair specific to diversifying antibody genes. This role is distinct from the known roles of PARP-1 in high-fidelity DNA repair, suggesting that the PARP-1 BRCT domain has a specialized role in assembling mutagenic DNA repair complexes involved in antibody diversification.

## Abstract 2

During affinity maturation, genomic integrity is maintained through specific targeting of DNA mutations. The DNA damage sensor PARP-1 helps determine whether a DNA lesion results in faithful or mutagenic repair.

## Author Summary

To produce a limitless diversity of antibodies within the constraints of a finite genome, activated B cells introduce random mutations into antibody genes through a process of targeted DNA damage and subsequent mutagenic repair. At the same time, the rest of the genome must be protected from mutagenesis to prevent off-target mutations which can lead to the development of lymphoma or leukemia. How antibody genes are specifically targeted is still largely unknown. A potential player in this process is the DNA-damage-sensing enzyme PARP-1, which recruits DNA repair enzymes to sites of damage. Using a chicken B cell lymphoma cell line because it has only a single PARP isoform and constitutively mutates its antibody genes, we compared the types of mutations accumulated in PARP-1^−/−^ cells to wild type. We found that in cells lacking PARP-1, the major pathway of mutagenic repair was disrupted and fewer mutations than normal were introduced into their antibody genes. To identify what might be important for mutagenesis, we tested different factors for their ability to rescue this mutagenic deficiency and found a role for the BRCT (BRCA1 C-terminal) domain of PARP-1, a consensus protein domain known to be involved in directing protein-protein interactions. Our evidence suggests that PARP-1 may be interacting with another hypothetical protein via its BRCT domain that is required for the mutagenic rather than faithful repair of DNA lesions in the antibody genes.

## Introduction

The generation of high affinity antibodies through affinity maturation in B cells relies on the introduction of mutations into expressed immunoglobulin (*Ig*) gene alleles by somatic hypermutation (SHM) or gene conversion (GCV). These closely related processes are mediated through introduction of a DNA lesion by activation-induced cytidine deaminase (AID), followed by fixation of a mutation at or nearby the damage site via a mutagenic, rather than the usual conservative, DNA repair mechanism [1],[2]. Mutations must be restricted to the *Ig* genes to protect the rest of the genome from accumulating potentially dangerous mutations, although this protection is far from perfect. Analysis of the mechanisms that direct mutagenesis to *Ig* loci has revealed the existence of multiple layers of regulation. One level of control is temporal regulation of expression of AID to activated B-cells in germinal centers, where cells with non-beneficial mutations can be quickly eliminated [3]. Another level of control is targeting of AID-mediated deamination to expressed *Ig* loci and, less frequently, a subset of other expressed genes through an as yet undefined transcription-dependent mechanism [4],[5]. A third level of control is the *Ig*-specific targeting of mutagenic repair. While identical lesions at non-*Ig* loci are usually repaired by a high-fidelity mechanism, at *Ig* loci, a mutagenic repair pathway predominates, either through translesion synthesis by error-prone polymerases or GCV [6].

While mutagenesis is necessary for high affinity antibody production, mistargeting of either the AID-mediated deamination events or the mutagenic repair of incidental mutations has been linked to the generation of B-cell lymphomas and leukemias through the introduction of mutations into tumor suppressors and proto-oncogenes such as *Bcl6*, *Myc*, *RhoH*, *Pim1*, and *Pax5* [7],[8],[9]. Recent data suggest that mistargeting of mutations occurs more frequently than previously thought, highlighting the importance of understanding how the processes that induce these mutations are targeted to specific genetic loci [6],[10],[11]. However, insights into the biochemistry through which either DNA lesions or mutagenic repair are targeted have been difficult to achieve, and so far have been limited to the definition of cis-acting DNA elements required for active mutagenesis at *Ig* loci [12],[13].

The enzyme PARP-1acts as a gatekeeper of DNA repair. It is one of the first proteins to respond to DNA damage, where it binds and recruits the appropriate DNA repair enzymes. There is a slower, background level of repair in PARP-1 deficient cells, but DNA repair is severely impaired and these cells are rendered hypersensitive to DNA damaging agents such as methyl methane sulfonate (MMS), N-Methyl-N′-Nitro-N-Nitrosoguanidine (MNNG), and ionizing radiation [14],[15],[16],[17]. In addition to a well-established role in base excision repair (BER), there is evidence that suggests that PARP-1 may also play a role in repairing double strand breaks, although whether by homologous recombination (HR), non-homologous end joining (NHEJ), or micro-homology mediated end joining (MMEJ) is still the subject of lively debate [14],[18],[19],[20],[21].

A potential clue to the mechanisms of mutation targeting has been suggested by a recent report that the enzyme PARP-1 is constitutively bound to a DNA sequence within the *Bcl-6* gene [22]—a locus which is frequently the subject of off-target mutations in B-cells [9],[23]. This observation prompted us to evaluate mutation targeting to the *Ig* loci in a PARP-deficient variant of chicken DT40 B-cell line, in which *Ig* loci are constitutively mutated via GCV. Remarkably, we observe a nearly complete loss of GCV at *Ig* loci in PARP-deficient cells that is independent of the rate of AID-induced DNA lesioning. Functional analysis of PARP-1 variants demonstrated that PARP-1 is necessary for repair of AID-mediated DNA lesions, and that the capacity of PARP-1 to support GCV requires its BRCT domain which, to our knowledge, has no previously characterized function.

## Results

The recent observation that PARP-1 is constitutively bound at the *Bcl-6* locus, a gene which is frequently the subject of mistargeted mutations in B-cells, prompted us to explore the role of PARP-1 in targeting mutations to *Ig* loci. Mice and humans have two PARP proteins that respond to DNA damage (PARP-1 and PARP-2) [20],[24]; while the murine knockout of PARP-1 is viable, the double knockout is embryonic lethal at day 8 [25], hindering use of murine systems for study of PARP function. Even so, a recent study of PARP-1^−/−^ mice revealed defects in T-cell dependent B-cell responses, decreased AID expression in activated B cells, and defects in antibody production [26], although, as previously reported, SHM and antibody production are not absent altogether [27]. However chickens have only one PARP enzyme that responds to DNA damage, allowing a complete PARP^−/−^ phenotype to be achieved via targeted knockout of the gene encoding PARP-1 in the chicken DT40 B-cell line [14].

### Inactive PARP-1 Mutant Blocks Repair of AID-Induced Lesions

**Figure 1:**
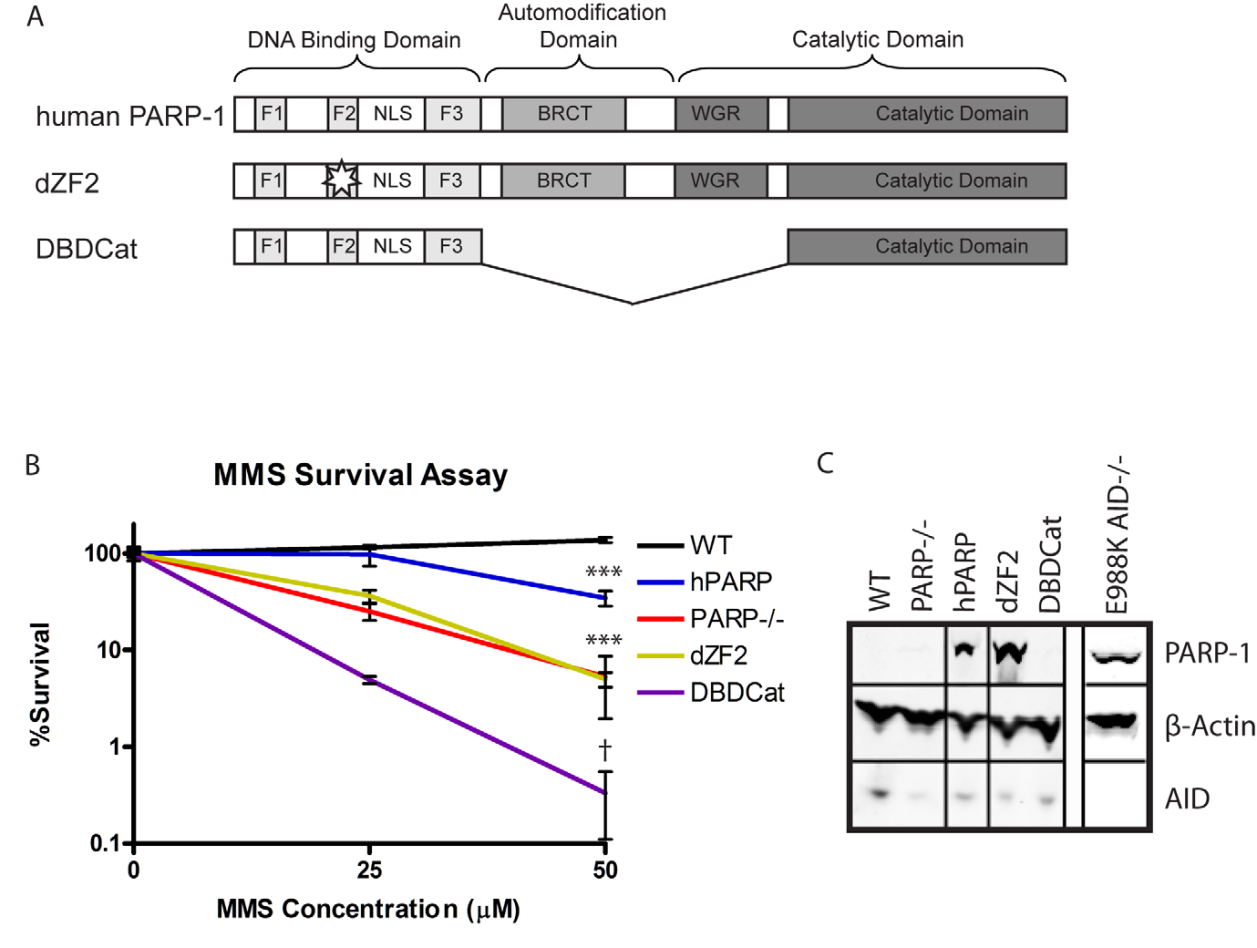
Functional effects of expression of human PARP-1 variants on survival in response to MMS-induced DNA damage. (A) Schematic of domains of human PARP-1 and variants. The functional domains of PARP-1 consist of a DNA binding domain (DBD), automodification domain (AMD), BRCT protein interaction domain (BRCT), and WGR/catalytic domain (WGR/Cat). The DBD contains 3 zinc finger domains, which are unusual in that they have specificity for DNA structure rather than sequence and recognize single strand breaks (SSBs) or double strand breaks (DSBs) [39],[40]. The AMD contains the lysine residues that act as poly-ADP-ribose (PAR) acceptors [35]. The WGR/catalytic domain catalyzes PAR formation when the DBD is bound to DNA, and PARylation of the AMD is thought to serve as a signal to recruit DNA repair enzymes such as XRCC1 as well as facilitates the release of PARP-1 from the site of DNA damage [41]. The BRCT protein interaction domain is of unknown function, as it has been shown to be dispensable for PARP-1's DNA repair functions in previous analyses [16]. hPARP: full length human PARP-1; dZF2: C125Y and C128Y mutations to prevent folding of the second zinc finger domain; DBDCat: DNA binding domain fused to a non-functional portion of the catalytic domain. (B) MMS survival assay comparing survival of the PARP-1 variants to MMS-induced DNA damage. Survival is measured by the ability to proliferate after 1 h of exposure to MMS at the indicated concentration. The experiment was performed in triplicate; error bars represent SEM. *** PARP-1^−/−^, dZF2, and hPARP *p*<.0001 compared to WT; PARP-1^−/−^ *p*<.0003 compared to hPARP; † *p*<.0001 compared to WT, *p* = .021 compared to PARP-1^−/−^; between PARP-1^−/−^ and dZF2 there is no significant difference. (C) Western blot showing levels of variant PARP-1 and AID expression with β actin as a loading control.

PARP-1 is thought to be one of the first proteins to respond to DNA strand breaks, where it binds and recruits the appropriate DNA repair enzymes [14],[18],[19],[20],[21]. Consistent with this model, PARP-1 deficient DT40 cells are rendered hypersensitive to DNA damaging agents such as MMS, MNNG, and ionizing radiation [14],[15],[16],[17]. To further define the parameters of PARP-1's role in these repair processes, we evaluated the sensitivity of PARP-1^−/−^ DT40 cells to MMS exposure, and assessed the capacity of human WT PARP-1 (hPARP), a DNA binding mutant of hPARP-1 (dZF2), and two enzymatically inactive variants of hPARP-1 (DBD-CAT and E988K) to restore survival upon MMS challenge (Figure 1A and B).

As expected, hPARP expression fully restores MMS resistance to the PARP-1^−/−^ cells, while the PARP-1 mutant lacking a DNA binding domain due to mutations in the cysteines critical for zinc finger folding is similar in phenotype to the knockout. In contrast, a catalytic inactive PARP-1 mutant that contains only the DNA binding domain and the catalytic domain minus the WGR portion exhibited poorer survival than the PARP-1^−/−^ cells (Figure 1B). As previously shown, this is likely due to the aggregation of inactive PARP-1 molecules at the site of DNA damage which could block access of DNA repair enzymes to the damaged site and/or deplete free PARP-1 and prevent binding and recruitment of repair enzymes to other sites of damage [28].

Surprisingly, we found that even after repeated attempts in parallel with successful transfection of other PARP-1 variants, we could not reconstitute PARP-1^−/−^ cells with a full length, inactive variant of PARP-1 (E988K), and thus we could not analyze the phenotype of PARP-1^−/−^ cells reconstituted with that variant. As this variant had been successfully expressed in mouse embryonic fibroblasts previously [28], we reasoned that a major difference between the DT40 cell context and other cell lines is the presence of constitutive AID expression and diversification of the *Ig* loci in DT40s. We therefore attempted expression of PARP-1(E988K) in both wild type and AID-deficient DT40 cells. Remarkably, we were unable to grow out any resistant clones in wild type DT40 cells, but clones stably expressing E988K readily grew from the parallel transfection of AID^−/−^ DT40s, with the transfection yielding dozens of transformants of which 15 were subjected to further analysis to verify expression (Figure 1C shows a representative clone). When we subsequently attempted to reconstitute E988K, AID^−/−^ DT40 clones with AID in the 4/TO vector (Invitrogen) with zeocin selection, of the 4 clones which eventually grew in the selective media, all had downregulated expression of E988K PARP-1 to below our limits of detection (as detected by Western blot, unpublished data). This dramatic selection against clones that coexpress AID and E988K suggested that PARP-1 plays an important role in the repair of lesions induced by AID.

### PARP-1^−/−^ Cells Are Deficient in GCV at *IgL* and *IgH*

**Figure 2:**
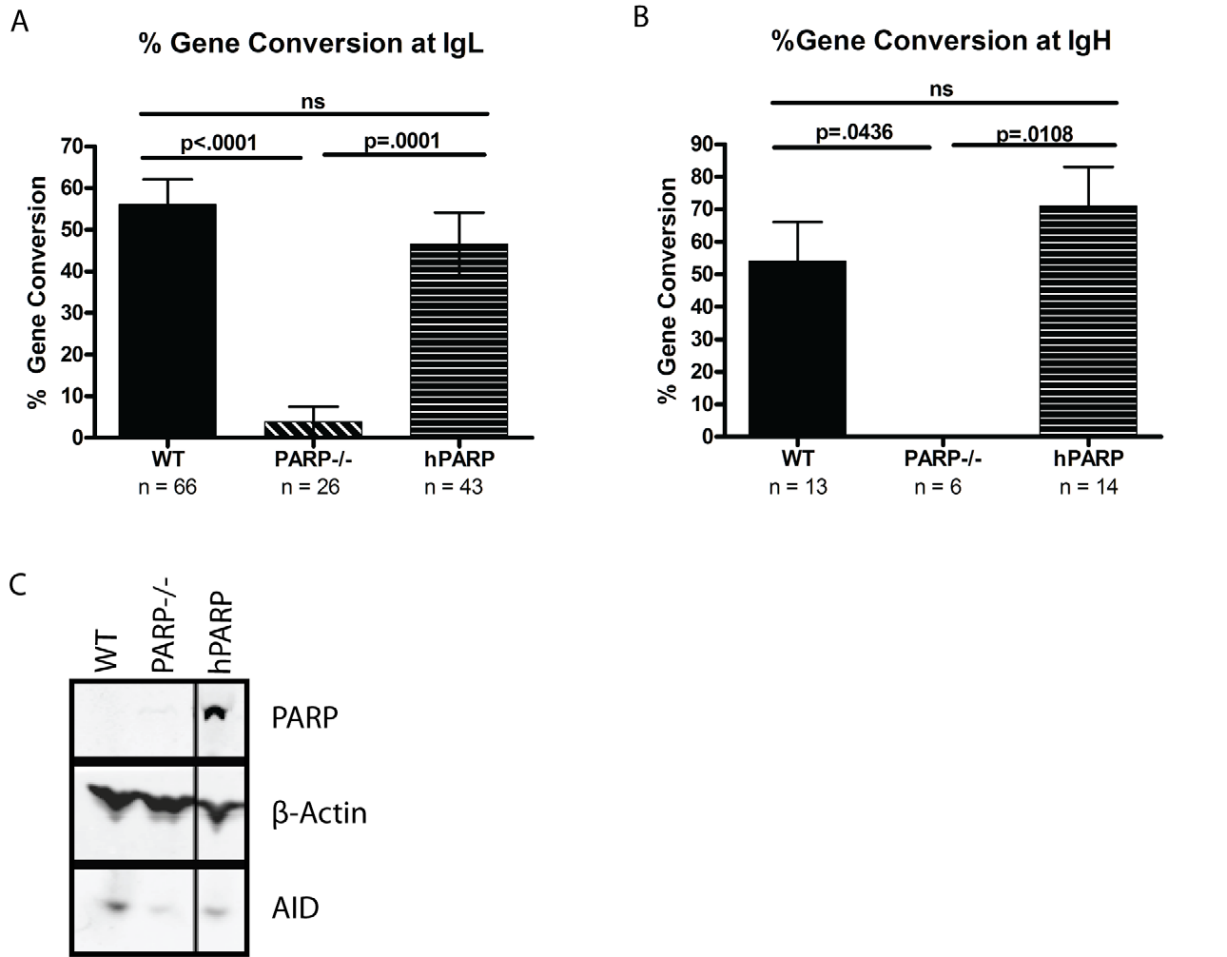
PARP-1 is required for Ig gene conversion. Gene conversion frequencies (+/− SEM) are indicated as a percentage of total mutations at IgL (A) and IgH (B). *n* = total number of mutations analyzed for each cell line at each locus. (C) Western blot showing PARP-1 and AID expression levels with β actin as a loading control.

The sensitivity of DT40s expressing AID to expression of PARP-1 E988K suggested that PARP-1 has a requisite role in repair of AID-induced lesions, either at mutating *Ig* loci or off-target lesions genome-wide. To assess the function of PARP-1 at mutating *Ig* loci, we sequenced the variable regions of the *Ig* light and heavy chains in PARP-1^−/−^ and WT cells. While mutations still detectably accumulated within *Ig* genes, although at a dramatically reduced rate, the PARP-1^−/−^ cells had essentially no discernible GCV events (*p*<.0001 at IgL and *p* = .0436 at IgH, Figure 2A and B). To confirm that the observed defect in GCV was due to the actions of PARP-1, we examined a cell line reconstituted with human PARP-1 (hPARP) (Figure 2C) and found that hPARP restored the GCV frequencies to wild type levels or above at both IgL (*p* = .0001) and IgH (*p* = .0108) (Figure 2A and B), suggesting that PARP-1 not only is required to repair AID-mediated deaminations but also influences the outcome of the resulting repair.

In this analysis, all mutations that matched chicken *Ig* pseudogene sequences published in the NCBI public database were counted as GCV events. This includes a subset of “ambiguous” mutations, which match the pseudogenes but occur as a single nucleotide change such that we cannot exclude the possibility that it arose as a point mutation. While the work of Saberi et al. shows that these mutations are generally true GCV events [29], to ensure that the process of classifying GCV events is not affecting our conclusions, we have also analyzed the data with the “ambiguous” mutations excluded from the analysis or scored as point mutations (Figure S2). In these additional analyses, the difference in GCV frequencies between WT and PARP−/− cells at the IgL locus remains highly significant (*p*<.0001 and *p* = .0006, respectively), although the number of mutations scored at the less well defined IgH locus was not sufficient to reveal a significant difference in these more conservative analyses (*p* = .1023 and *p* = .1280, respectively). Subsequent restoration PARP-1 expression restores GCV at both IgL (*p* = .0002 and *p* = .0029) and IgH (*p* = .0108 and *p* = .0108). The individual mutations observed at IgL can be found in Figure S1 and a schematic of mutations observed at IgH is shown in Figure S2E.

### AID Overexpression Does Not Restore GCV to PARP-1^−/−^ Cells

**Figure 3:**
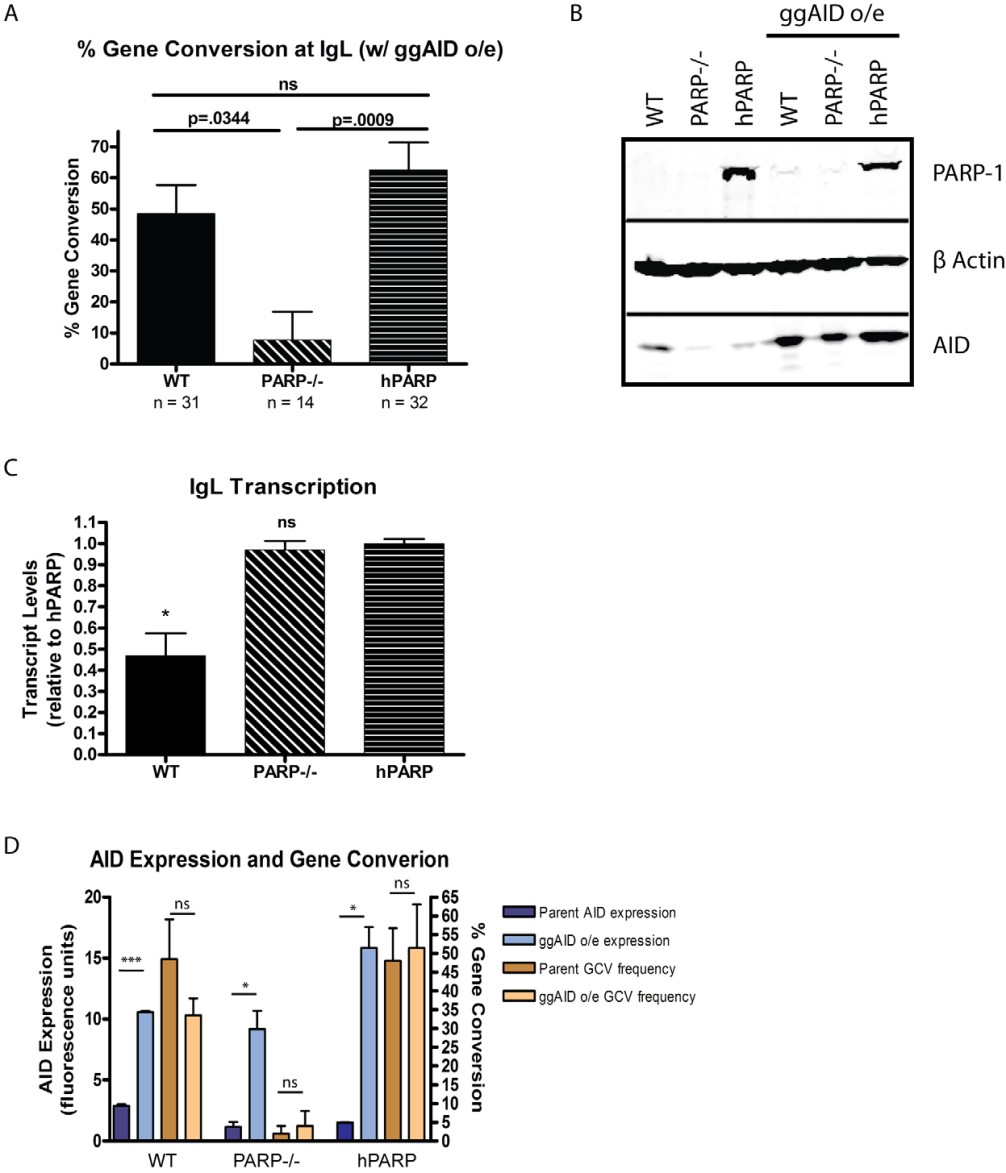
AID overexpression does not restore GCV to PARP-1^−/−^ cells. (A) Gene conversion frequencies (+/− SEM) in cell lines overexpressing ggAID. *n* = total number of mutations analyzed for each cell line. The total number of sequences analyzed was 169 WT, 137 PARP-1^−/−^, and 106 hPARP. (B) Western blot showing increase in AID expression upon transduction with ggAID retrovirus. (C) *IgL* transcript levels (mean +/− SEM) are similar in cell lines which do and do not support GCV. * *p*<.05, *ns* = not significant compared to hPARP. (D) AID expression levels do not directly influence GCV frequencies. Blue bars are AID expression levels (mean +/^−^ SEM) before (dark blue) and after (light blue) transduction with ggAID cDNA as measured by Western blot and quantified by LICOR Odessey infrared imaging, normalized to β actin. Brown bars are GCV frequencies (mean +/− SEM) before (dark brown) and after (light brown) transduction with ggAID cDNA as a percentage of total mutations observed for the indicated cell lines. *** *p*<.0001, * *p*<.05, *ns* = not significant.

In the course of characterizing the GCV of PARP-1^−/−^ cells, we observed that the PARP-1^−/−^ cell line and its hPARP derivative express less AID than the WT DT40 cells, and that the PARP-1^−/−^ and hPARP lines had a lower overall mutation rate. As low AID expression seemed likely to be the inadvertent result of selection during derivation of the parental PARP-1^−/−^ clone that was subsequently carried over to the reconstituted hPARP cell line, we investigated whether the reduction in AID expression and corresponding overall mutation rate could be influencing the proportion of GCV events. For this purpose, we used a retroviral vector to overexpress gallus gallus AID in each cell line, and matched AID expression as well as *IgL* transcript levels in selected clones (Figure 3B and C), as the rate of target gene transcription has also been shown to affect mutation rate [30],[31],[32]. Consistent with previous reports, overexpressing AID does not significantly increase the proportion of mutations which are GCV events [29] and does not restore GCV to the PARP-1^−/−^ cells, in spite of increasing the AID expression to well above the original WT levels (Figure 3A and D). Interestingly, overexpression of AID does not significantly increase the mutation rate in the PARP-1−/− cells as it does in hPARP, and may be revealing a dose-dependent effect of PARP-1's ability to mediate GCV repair of deamination events as increasing AID expression results in a WT GCV phenotype intermediate to PARP-1^−/−^ and hPARP (Figure 3A). Furthermore, our ability to generate high stable expression of AID in PARP-1^−/−^ cells suggests that the reduced mutation rate in PARP-1^−/−^ cells relative to hPARP reconstituted cells cannot be explained by loss of cells that sustain AID-mediated lesions, but rather may reflect a reduced rate of mutagenic repair of AID-mediated lesions.

### Treatment with the HDAC Inhibitor Trichostatin A Does Not Rescue GCV in PARP-1 Knockouts

**Figure 4:**
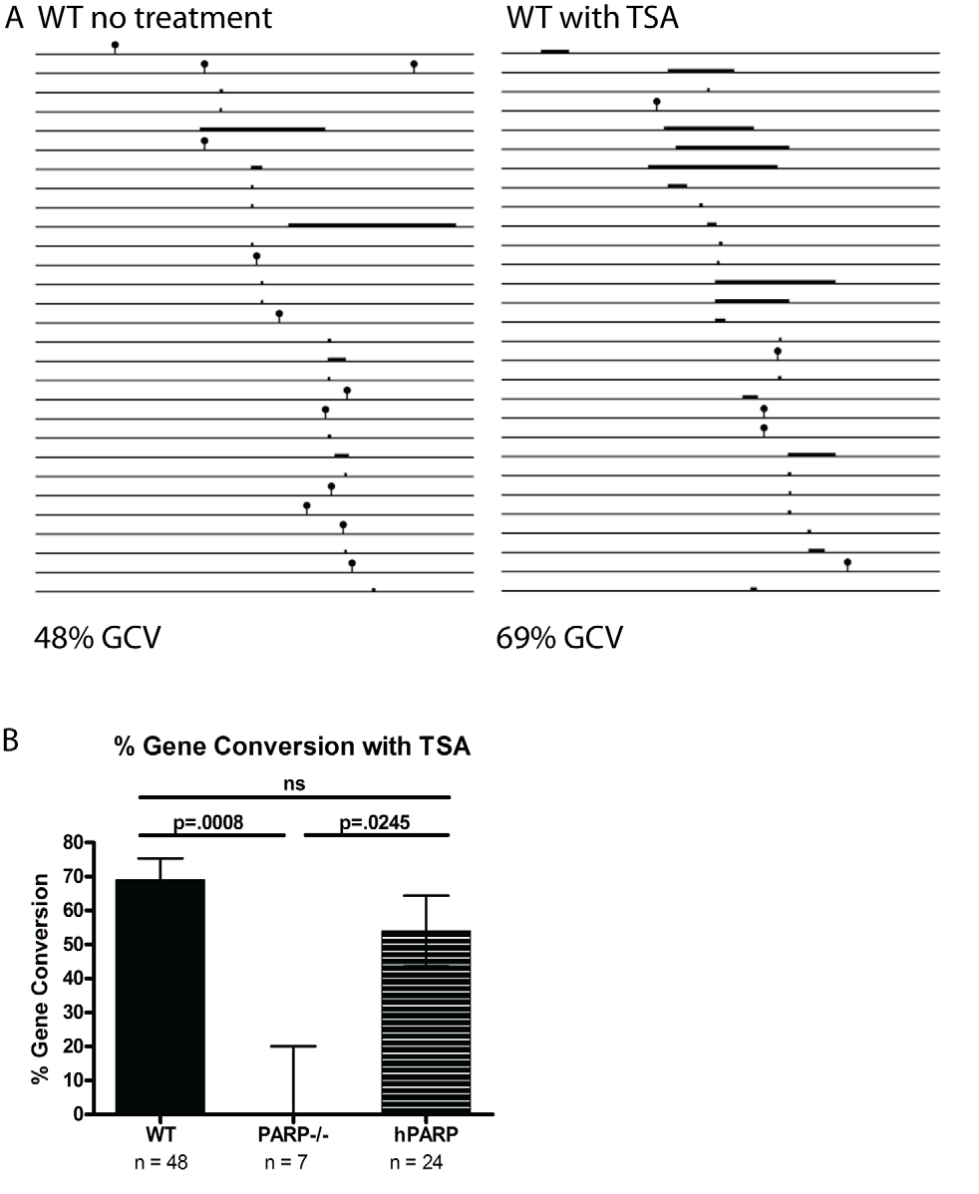
Culture in TSA increases GCV rates at IgL in a PARP-1-dependent fashion. (A) Line drawing depicting point mutations (lollipops) and gene conversion events (bars) at WT *IgL* in the absence and in the presence of TSA. (B) Proportion of total mutations that are GCVs in TSA treated cultures. Error bars are (+/−) SEM. *n* = total number of mutations observed for each cell line.

As an initial step towards defining the role of PARP-1 in repair of AID-induced lesions, we investigated the influence of chromatin accessibility on mutagenic repair at *Ig* loci. It has been proposed that PARP-1 modifies histones at the site of DNA damage to open chromatin and increase the accessibility of a damaged site to repair enzymes [33]. To determine whether altered chromatin accessibility could account for deficient GCV in PARP-1^−/−^ cells, we cultured PARP-1^−/−^ cells in trichostatin A, a histone deacetylase (HDAC) inhibitor, as this has previously been shown to increase GCV rates, presumably via increasing accessibility of the pseudogene repair templates to repair machinery [34]. While the percent and length of GCV events in WT cells went up dramatically, indicating that the treatment was effective (Figure 4A), the PARP-1^−/−^ cells are still unable to carry out GCV (Figure 4B). Thus, the mechanism of PARP-1's role in promoting GCV at *Ig* loci is not through increasing chromatin accessibility.

### PARP-1 Does Not Promote HR Repair of DSBs

**Figure 5:**
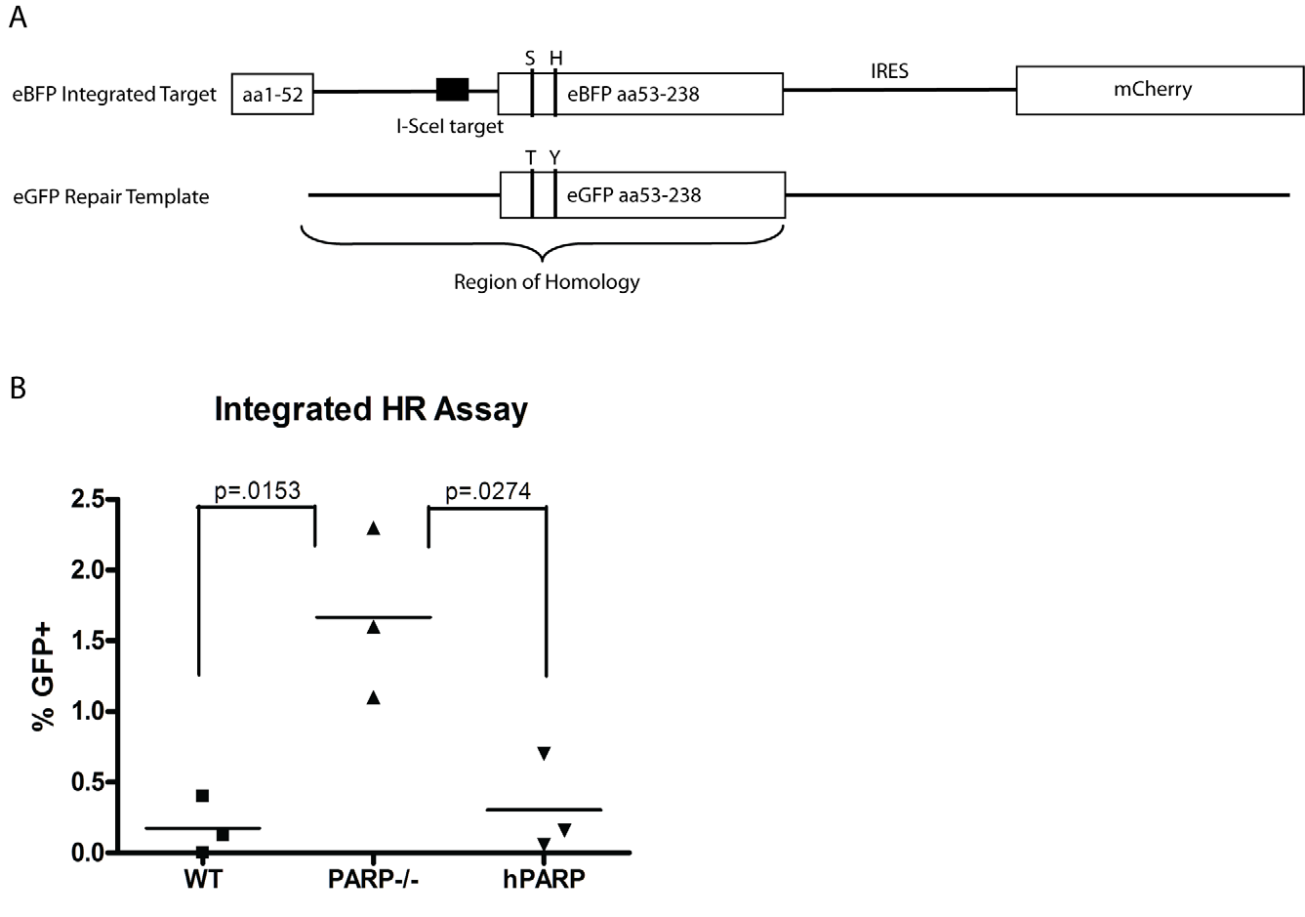
PARP-1 does not promote HR in an integrated reporter assay. (A) Schematic of HR reporter assay. eBFP target containing I-SceI target sequence is integrated into host genome. I-SceI and repair template are transfected into cells. HR repair of DSB is detected by GFP fluorescence measured by flow cytometry. (B) Percent GFP positive cells normalized for transfection efficiency in indicated cell lines 48 h after transfection of I-SceI and repair template. Data points represent independent experiments. There is no significant difference between WT and hPARP.

*Ig* GCV in DT40s proceeds through a pathway involving HR. Since PARP-1 has previously been implicated in HR repair, we assessed whether PARP-1's capacity to promote *Ig* GCV was a part of a general role for PARP-1 in promoting HR by evaluating the capacity of PARP-1^−/−^ cells to mediate HR in response to a single DNA double strand break generated by the homing endonuclease I-SceI (Figure 5A). In a single-copy, integrated assay of HR, we find that the expression of PARP-1 does not promote HR. Rather, in agreement with studies by Wang et al., PARP-1 may suppress HR and promote alternate DNA repair pathways (Figure 5B) [19]. This result indicates that the activity of PARP-1 at *Ig* loci is not part of a global role promoting HR, and further supports the hypothesis that PARP-1 has a specific role within GCV as a mutagenic repair pathway operating at *Ig* loci. It also raises the question of whether it is best to consider GCV at *Ig* loci as an unusual, mutagenic instance of otherwise high-fidelity HR or if a better model would be to consider GCV one pathway for mutagenic repair at *Ig* loci that uses much, but not all, of the same repair machinery as HR.

### BRCT Domain of PARP-1, While Dispensable for Global DNA Repair, Is Required for *Ig* GCV

**Figure 6:**
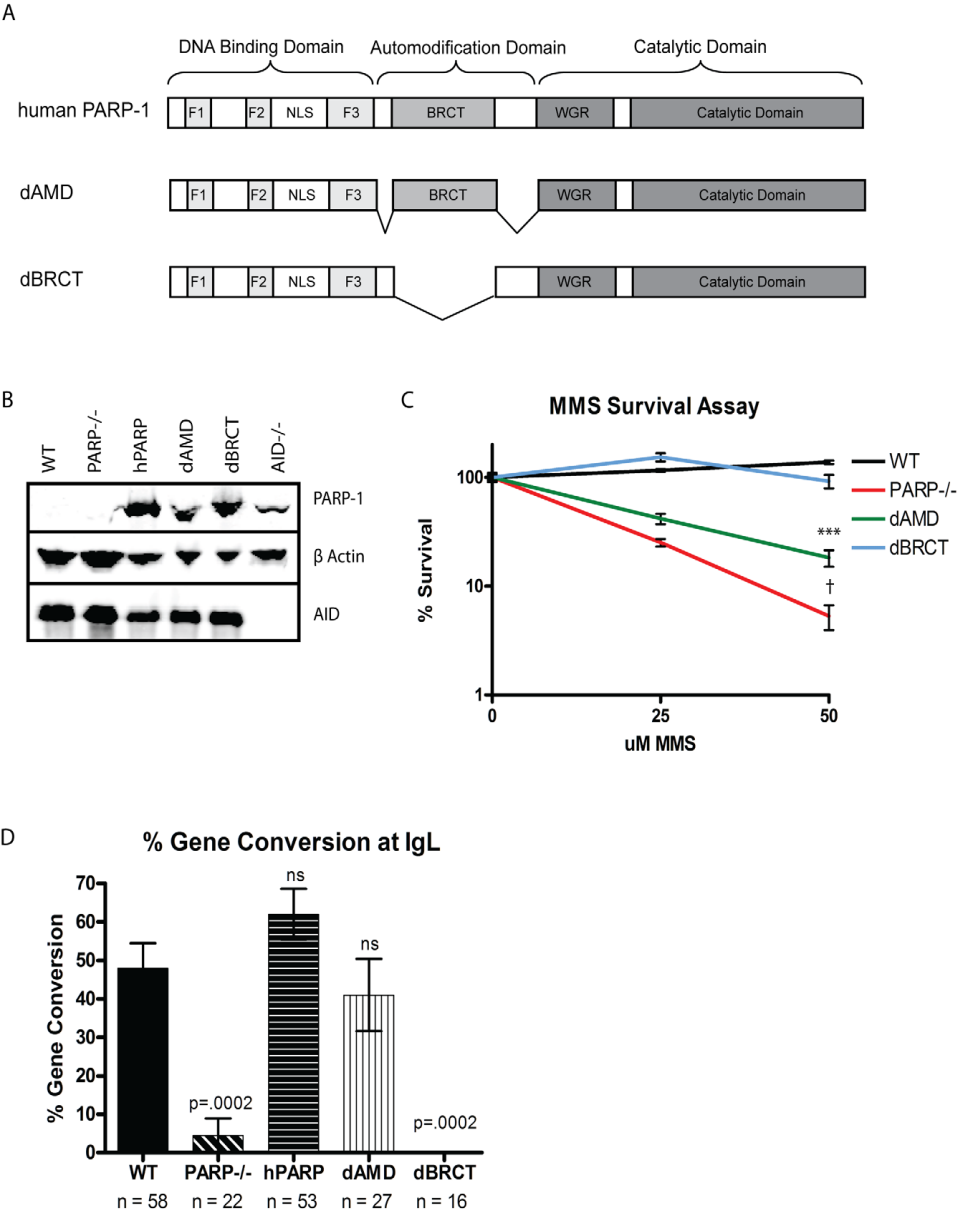
The PARP-1 BRCT domain is required for immunoglobulin gene conversion. (A) Schematic of the domains of PARP-1 and variants dAMD and dBRCT. (B) Western blot showing levels of PARP-1 and AID expression with β actin as a loading control. (C) Survival of PARP-1 variants to MMS-induced DNA damage. Experiment was performed in triplicate and error bars represent SEM. *** *p*<.0001 compared to WT or dBRCT; † *p*<.0001 compared to WT or dBRCT, *p* = .02 compared to dAMD. There is no significant difference between WT and dBRCT. (D) Frequencies of gene conversion events as a proportion of total mutations at the *IgL* locus (+/− SEM). *n* = total number of mutations analyzed for each cell line.

To further investigate the mechanism by which PARP-1 mediates GCV, we evaluated the role of specific PARP-1 subdomains in promoting mutagenic repair at *Ig* loci. Hypothesizing that the role of PARP-1 in mutagenic repair at *Ig* loci may be distinct from its established role in high-fidelity BER, we compared DNA BER and *Ig* GCV among PARP-1^−/−^ clones reconstituted with PARP-1 variants containing deletions or inactivating mutations in each domain. Consistent with their lack of ability to reconstitute any detectable DNA BER (see Figure 1), sequence analysis of *Ig* loci in PARP-1^−/−^ cells reconstituted with the dZF2 or DBD-CAT variants indicated that expression of either of these PARP-1 variants was unable to reconstitute Ig GCV (Figure S3). We then analyzed PARP-1 variants that retained significant capacity to reconstitute BER in the PARP-1^−/−^ cells. Analysis of PARP-1^−/−^ cells reconstituted with variants of PARP-1 containing either a deletion of the BRCT domain (dBRCT lacks aa384–479) or the automodification domain sparing the BRCT domain (dAMD lacks aa372–383 and 480–524) (Figure 6A) gave surprising results. In an MMS survival assay, the dBRCT cells survived as well as WT cells (Figure 6C), consistent with previous data from our lab and others indicating that the BRCT portion of the automodification domain is not required for PARP activation via base-damaging agents or PARP-dependent base excision repair [16],[35]. In the same assay, dAMD-expressing cells exhibited an intermediate survival phenotype when treated with MMS (Figure 6C), consistent with the dAMD mutant exhibiting a significant but delayed capacity to catalyze NAD degradation and ADPR production in response to base damaging agents [16]. We then assayed the ability of these cell lines to undergo GCV at *IgL* and found that the capacity to mediate GCV does not correspond with the ability of the cells to carry out global high-fidelity DNA repair. In contrast, sequence analysis of *Ig* loci revealed that the dBRCT cells, like PARP-1^−/−^ cells, were essentially unable to mediate GCV and had a correspondingly low overall mutation rate, whereas the dAMD cells were able to gene convert at *IgL* at or near WT levels (Figure 6D and Figure S3). This observation demonstrates that the role of PARP-1 in mutagenic GCV at *Ig* loci is distinct from its role in high-fidelity DNA BER. Furthermore, it implies a novel role for the BRCT domain of PARP-1 in mediating mutagenic DNA repair at *Ig* loci.

### Independent of AID Expression, PARP-1^−/−^, and dBRCT Variants Accumulate Mutations at a Lower Rate than WT or hPARP

**Figure 7:**
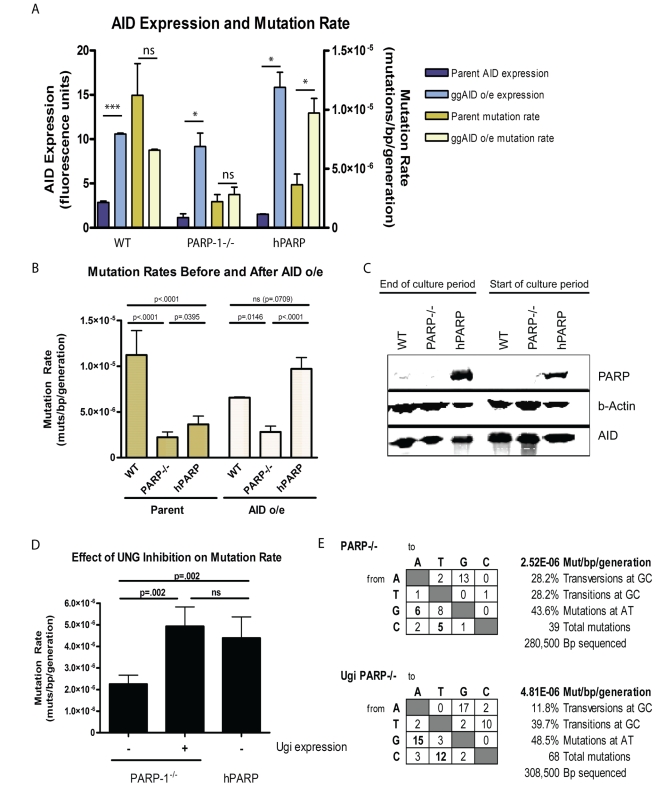
AID-induced lesions are more likely to undergo high-fidelity repair in PARP-1^−/−^ cells. (A) Mutation rate is limited in PARP-1^−/−^ cells, even when AID expression is restored. Blue bars show AID expression levels (mean +/− SEM) before (dark blue) and after (light blue) transduction with ggAID cDNA as measured by Western blot and quantified by LICOR Odessey infrared imaging, normalized to β actin. Yellow bars show total mutation rates (mean +/− SEM) before (dark yellow) and after (light yellow) transduction with ggAID cDNA, measured as total mutations/bp/generation for the indicated cell lines. *** *p*<.0001, * *p*<.05, *ns* = not significant. (B) Increased AID expression restores mutation rate in hPARP to WT levels, but PARP-1^−/−^ remain reduced. (C) Western blot showing PARP-1, AID, and β-actin levels at the beginning and the end of the culture period. (D) Mutation rate (mean +/− SEM when available) in PARP-1^−/−^ cells with and without UGI expression, compared to hPARP cells. (E) Tables showing the distribution of mutations in PARP-1^−/−^ and PARP-1^−/−^UGI cell lines. The changes are scored from the nucleotide indicated on the left to the nucleotide indicated on the top of the table.

When AID is overexpressed in cells overexpressing PARP-1 (hPARP), we observe that the mutation rate increases accordingly. However, when AID is overexpressed in PARP-1^−/−^ cells, we do not see a significant change in mutation rate. This suggests that while AID deamination is the initial rate-limiting step for SHM and GCV, expression of PARP-1 may further limit mutation rate during repair of these lesions (Figure 7A and B). One potential explanation for these observations is that lack of PARP-1 selects against PARP-1^−/−^ cells with high AID expression that would have accumulated mutations. To test this possibility, we evaluated the stability of AID overexpression over the 3 wk culture period used for accumulation of mutations, based on the rationale that selection pressure against mutations would lead to a decreased expression of AID. In this experiment, comparison of AID expression before and after 3 wk of culture revealed no detectable difference in AID expression, indicating that selection against the PARP-1^−/−^ cells that would have accumulated mutations cannot account for the observed decrease in mutation rate in the PARP-1^−/−^ cells (Figure 7C). Thus, the observed lower mutation rate reflects either a decreased rate of DNA lesioning or an increased rate of faithful repair of AID-mediated DNA lesions. While the combined evidence of an established role for PARP-1 in DNA repair and the baseline level of mutations which continue to accumulate in the PARP-1^−/−^ cells lead us to prefer the latter hypothesis, we sought to resolve this issue by expressing an UNG inhibitor UGI in the PARP-1^−/−^ DT40 cells expressing endogenous levels of AID. UGI has been shown to reveal an “AID footprint” of activity by blocking BER of deaminated cytosines and increasing the relative frequency of mismatch repair mutations at A/T and “replication over” events, fixing C to T and G to A mutations at the site of deamination [36]. Correspondingly, we found that UGI expression results in decreased transversion mutations at G/C base pairs, and a relative increase in replication over errors at G/C and mismatch repair mediated mutations at A/T base pairs (Figure 7E). Analyzing the rate of mutation by sequence analysis, we observed that UGI expressing PARP-1^−/−^ cells exhibited an increased rate of mutation relative to their parent cell line, and now matched the mutation rate observed in the hPARP cells (Figure 7D), chosen as a control because they express a similar level of AID (see Figure 2). These findings support our hypothesis that the low mutation rate of the parental cell line was not the result of decreased deamination events, but rather reflected an increased proportion of high-fidelity repair. This collection of evidence leads us to conclude that PARP-1 is promoting mutagenic repair at *Ig* loci and that AID-induced lesions are more likely to be repaired faithfully in the PARP-1^−/−^ cells.

## Discussion

In this paper, we studied the role of PARP-1 in mediating repair of AID-induced lesions at diversifying *Ig* loci. By sequencing *Ig* light and heavy chain genes in PARP-1^−/−^ DT40 B-lymphocytes, we found that the overall mutation rate is reduced and GCV is essentially eliminated in the absence of PARP-1, and these defects can be fully reconstituted by expression of the hPARP-1 gene. Dissection of the biochemical mechanisms underlying PARP-1's involvement in *Ig* diversification demonstrated that PARP-1 DNA binding and BRCT protein-protein interaction domain are required, while the major site of automodification is not. Furthermore, while the overall mutation rate in PARP-deficient cells could be slightly increased by AID expression, restoration to WT levels required concomitant inhibition of uracil-DNA-glycosylase, suggesting that PARP-deficiency leads to an increased rate of high-fidelity repair at *Ig* loci through the UNG-dependent BER pathway. Taken together, our data suggest that PARP-1 is an important part of the biochemical processes that promote mutagenic repair over faithful repair at *Ig* loci, through a mechanism that requires an intact BRCT domain.

The requirement for the BRCT domain of PARP-1 in promoting mutagenic repair at *Ig* loci revealed in our studies defines a novel role for the BRCT domain of PARP-1. In spite of the extensive work which has lead to our current understanding of the subdomains of PARP-1, the function of the BRCT portion of the automodification domain has remained a mystery, as it is not required for PARP-1 mediated repair of the other types of DNA damage which have been studied [16],[35]. As BRCT domains are thought to function as protein-protein interaction domains, an intriguing possibility arising from our observations is that there may be a protein interaction partner of the PARP-1 BRCT domain which is involved in the mutagenic repair of AID-induced lesions but not involved in high-fidelity repair of other types of DNA damage. Identification of the hypothetical interaction partner(s) for the PARP-1 BRCT domain should further illuminate the mechanisms involved in PARP-1-dependent targeting and regulation of mutagenic DNA repair. PARP-1-dependent targeting of mutagenic repair may also account for the frequent mistargeting of mutations to the *Bcl-6* gene in B-cells, as PARP is constitutively targeted to the *Bcl-6* locus via sequence specific binding of its zinc fingers [37], and it would be interesting to see if PARP-1 also binds specifically to other common sites of mistargeted mutations involved in malignant transformation such as *Bcl-2*, *Pim1*, *Pax5*, *Myc*, or *RhoH*.

Diverse lines of evidence have recently developed to support the concept that AID-mediated DNA lesions are not uniquely targeted to *Ig* loci [10],[11] but rather that off-target deamination events in germinal center B-cells went undetected because those that occur outside the *Ig* loci primarily undergo high-fidelity repair [6]. This new information emphasizes the importance of understanding not just how AID is targeted to *Ig* loci but, equally as important, how mutagenic repair of deaminations is targeted to the *Ig* loci to protect B cells from the dangers associated with antibody diversification such as oncogenesis. Our data demonstrating a role for PARP-1 in both high-fidelity BER genomewide and mutagenic repair of deaminations at *Ig* loci present a mechanism for the targeting of mutagenic repair. PARP-1, known to play a key role in directing repair of alkylated DNA bases towards BER through interactions with the PARylated AMD, also directs repair down a mutagenic pathway at *Ig* loci through interactions with the BRCT domain.

**Figure 8:**
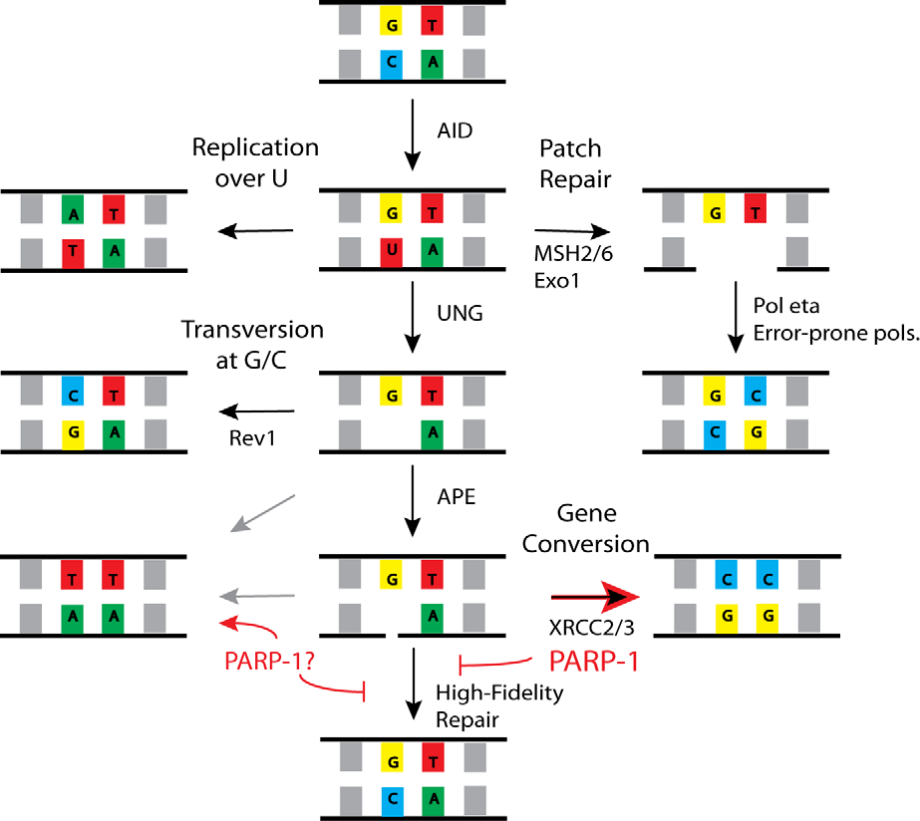
A model for the role of PARP-1 in GCV and SHM. PARP-1 increases GCV and decreases high-fidelity repair, although whether this is accomplished by inhibiting high-fidelity repair and thus diverting repair to a mutagenic pathway or by directly promoting GCV is not yet clear.

Our data suggest that in the WT system, PARP may be acting at the site of a single strand break generated by AP lyase to either (1) directly promote GCV repair of breaks that would otherwise undergo high-fidelity repair or to (2) prevent high-fidelity repair at Ig loci, resulting in diversion to the less efficient error-prone repair pathways, including GCV and SHM (Figure 8). When normal GCV pathways are blocked, such as in XRCC2/3 knockouts, the lesions are diverted to other mutagenic repair pathways, such that the total mutation rate is unchanged [38]. In PARP-1^−/−^ cells, the lesions enter a high-fidelity repair pathway rather than undergoing GCV, which is more consistent with the latter hypothesis.

On the other hand, an early report from PARP-1^−/−^ mice would favor the former hypothesis, as the authors failed to find a hypermutation defect in B cells from those mice [27]. However, in those experiments Jacobs et al. examined ex vivo germinal center B cells which had undergone intense selective pressure for the accumulation of mutations. As we have shown that SHM is not eliminated but reduced in the absence of PARP, and the reported number of mutations in the PARP-1^−/−^ mice was reduced by more than one third (although they did not report how many sequences were analyzed from each mouse), our results are not in contradiction to this early report, and PARP-1 could still play a role in inhibiting high-fidelity repair during SHM and GCV. Additionally, a subsequent report identified reduced T cell-dependent responses and reduced AID expression in PARP-1^−/−^ mice [26], which could be explained by increased cell death among hypermutating B cells consistent with the theory that PARP-1^−/−^ B cells accumulate fewer mutations than WT B cells, and that the cells which fail to increase affinity by hypermutation are selected against in germinal centers. Our results establish a role for PARP-1 in the repair of the AID-induced lesions required for SHM and GCV, and further show that this role is mediated in part by the BRCT domain of PARP-1, a domain with, to our knowledge, no previously known function. Without the BRCT domain of PARP-1, the mutation rate is lower and GCV essentially absent in DT40 B cells. The requirement for the BRCT domain of PARP-1 may suggest that an interaction partner for PARP-1 is important for mediating this role, likely by inhibiting high-fidelity repair in the UNG-dependent BER pathway and allowing alternative, mutagenic repair pathways to predominate or, alternatively, by directly promoting GCV, thus allowing fewer lesions to enter a high-fidelity repair pathway.

## Materials and Methods

### PCR and Sequencing of *IgL* and *IgH*

The variable regions of *IgL* and *IgH* were amplified with Accuprime Pfx and blunt end Topo cloned. Single colonies were picked and sequenced using the M13 reverse primer. The primers used for PCR were IgL-F: CAGGAGCTCGCGGGGCCGTCACTGATTGCCG, IgL-R: GCGCAAGCTTCCCCAGCCTGCCGCCAAGTCCAAG, IgH-F: CGGGAGCTCCGTCAGCGCTCTCTGTCC, IgH-R: GGGGTACCCGGAGGAGACGATGACTTCGG.

### Categorization of Mutations

A baseline rate of mutation was determined by sequencing an irrelevant gene, the constant region of IgL, or the variable region of IgL in AID−/− cells. The polymerases Pfx (Invitrogen) and Pfu Ultra (Stratagene) were also compared (Figure S4). The baseline mutation rates were well below the observed mutation rates in this study, and we decide to proceed with our analyses using Pfx Accuprime polymerase. Sequences were aligned using Phred and Phrap and viewed in Consed. High quality base discrepancies were noted and subjected to further analysis. As the total mutation rate was much lower than 1 mutation/read, tracks of multiple mutations in a read were scored as GCV events. Single mutations for which no donor template could be identified were scored as point mutations. To categorize ambiguous mutations (which match the pseudogene templates but occur in isolation), results were compared when these mutations were (1) excluded from the analysis, (2) always considered point mutations, and (3) always considered GCV events. These changes made little difference to the final analysis as the mutations in the PARP-1^−/−^ and dBRCT cell lines very rarely matched the pseudogene sequences through either blast searches or direct comparison to a database of collected pseudogene sequences and so were able to be clearly scored as point mutations. While the ability of WT DT40s to undergo GCV is not in question, in order to avoid missing any GCV events which may occur in the PARP^−/−^ and dBRCT cell lines, it was decided to use the most inclusive definition of a GCV event, which is every mutation that matches the pseudogene sequences (annotated as “similar to immunoglobulin lambda chain” within gi 118098819) by blastn. At *IgH*, where the pseudogenes are not well characterized, a match resulting from a whole genome blast which was located adjacent to *IgH* was considered a suitable donor sequence for a GCV event and again, and all mutations with a donor template match were scored as GCV events. In spite of the poor assembly at IgH, the availability of donor templates as assessed is equivalent for all the cell lines used, so they may be compared. *p* values were generated using Fisher's exact test.

### RT-PCR and Quantification of IgL Transcript

Q-PCR was performed on a BioRad icycler using the BioRad SYBR green master mix. The annealing temperature was 58°C. The following primers were used: IgL-F: caggagctcgcggggccgtcactgattgccg, IgL-R: gcgcaagcttccccagcctgccgccaagtccaag, Beta Actin F: tgagagggaaatcgtgcgtgacatc, Beta Actin R: caggaaagagggttggaacagagcc. IgL transcript level was normalized to β-actin and hPARP expression using the ΔΔCt method. Data analysis was performed using Microsoft Excel and Graphpad Prism.

### Western Blots

Whole cell lysates were separated by SDS-PAGE, transferred to Millipore immobilon membrane, and probed with the following antibodies: PARP-1: ALX-210-302 (Alexis Biochemicals), βActin: A2228 (Sigma-Aldrich), and AID: LS-C34861 (Lifespan Biosciences). Secondary antibodies were labeled with IRdye-700CW or IRdye-800CW and analysis and quantification was done on the LICOR Odyssey Infrared Imager.

### Tissue Culture

DT40 cells were cultured in RPMI with 10% FBS, 5% CS, Pen/Strep, and b-ME at 41°. PARP-1^−/−^ cells and the WT parent cell line were a generous gift from S. Takeda. Cell lines reconstituted with hPARP-1 and the variant hPARP constructs were generated by electroporation of PARP-1 cDNA in the 5/TO vector (Invitrogen) at 550V, 25 uF in 4 mm cuvettes using the GenePulser from Bio-Rad. Colonies which grew in hygromycin were matched for PARP-1 expression by Western blot. AID^−/−^ cells were a gift from JM Buerstedde. AID^−/−^ cells transfected with E988K were then transfected with human AID cDNA in the 4/TO vector (Invitrogen) as above, with selection in zeocin. Cells treated with TSA were incubated in 2 ng/mL TSA (Sigma T8552), refreshed daily, for the entire culture period. Cell lines overexpressing AID used for mutation analysis were generated by retroviral transduction of a plasmid encoding chicken AID IRES GFP, another gift from S. Takeda. GFP high cells were subcloned and AID expression was measured by Western blot. All cells used for mutation analysis were subcloned by limiting dilution immediately before the culture period to ensure a homogenous starting population. Cells were then allowed to accumulate mutations for a period of 6 wk in initial experiments and 4 wk for experiments in which all the cell lines overexpress AID. There were no notable differences in generation time and all cells were split 1∶16 every other day. For calculations which include generation time, 12 h was used.

### HR Assays

A recombination substrate encoding BFP containing an intron with the recognition sequence for I-SceI was transduced into DT40s using a limiting titer of lentivirus to bias toward single integration events (cultures with less than 5% transduction efficiencies were used). Transient transfection of a GFP repair template plus I-SceI expression plasmid was performed by electroporation at 250 V, 950 uF in 4 mm cuvettes. Parallel transfection of a GFP control plasmid was used to estimate transfection efficiency and frequency of HR was calculated as the percent of mCherry positive cells that were also GFP positive, divided by the percent that were positive for the GFP control.

### MMS Survival Assays

Cells were exposed to MMS at the indicated concentrations for 1 h at 37°. They were then washed 2× in fresh media and resuspended in 3 mL media. 450 ul of 3% agar was added and 1 mL was plated in triplicate. Plates were grown for 3–4 d at 41° before colonies were counted.

## Supporting Information

Figure S1 **Mutations observed in the IgL sequence of WT, PARP-1^−/−^, and hPARP cell lines.** Point mutations are indicated below the reference sequence in red. Gene conversion events are indicated in boxes. The total number of sequences analyzed to generate these data were 93 WT, 184 PARP^−/−^, and 186 hPARP, all after 35 d in culture (70 generations).
(0.24 MB PDF)

Figure S2 **Different assessments of GCV do not change the dependence on PARP-1.** Gene conversion frequencies at IgL when (A) percent gene conversion is calculated when “ambiguous” mutations (those that match upstream pseudogenes, but occur in isolation) are excluded from analysis or (B) when “ambiguous” mutations are categorized as point mutations. (C) and (D) are the same analyses at the IgH locus. (E) Line drawing depicting point mutations (lollipops) and gene conversion events (bars) in WT, PARP-1^−/−^, and hPARP at the *IgH* locus.
(0.75 MB TIF)

Figure S3 **Schematic representation of mutations observed at IgL in multiple PARP-1 variants.** Line drawing depicting representative point mutations (lollipops) and gene conversion events (bars) at *IgL* in each of the cell lines used in this study after 30 d in culture (60 generations). (A) WT, 58 total mutations in 168 reads. (B) PARP-1^−/−^, 22 total mutations in 175 reads. (C) hPARP, 53 total mutations in 106 reads. (D) dAMD, 27 total mutations in 79 reads. (E) dBRCT, 16 total mutations in 236 reads. (F) DBDCat, 23 total mutations in 255 reads. (G) dZF2, 26 total mutations in 430 reads.
(0.56 MB TIF)

Figure S4 **Background mutation rates of polymerase and DT40s in culture.** (A) Mutations accumulated in an irrelevant gene in DT40 cells over 8 wk (120 generations) in culture when amplified with Pfx (Invitrogen). (B) Mutations accumulated in the constant region of IgL in DT40 cells over 6 wk (84 generations) in culture when amplified with Pfx (Invitrogen). (C) Comparison of mutations accumulated in the variable region of IgL in AID−/− PARP^WT^ DT40 cells over 10 wk (140 generations) in culture when amplified with Pfx (Invitrogen) or Pfu (Stratagene). (D) Comparison of mutations accumulated in the variable region of IgL in Ugi expressing PARP-1^−/−^ DT40 cells over 3 wk (46 generations) in culture when amplified with Pfx (Invitrogen) or Pfu (Stratagene).
(0.02 MB PDF)

## Abbreviations

AID: activation induced cytidine deaminase
BER: base excision repair
GCV: gene conversion
HDAC: histone deacetylase
hPARP: human PARP-1
HR: homologous recombination
Ig: immunoglobulin
MMS: methyl methane sulfonate
MNNG: N-Methyl-N′-Nitro-N-Nitrosoguanidine
NHEJ: non-homologous end joining
PARP-1: poly-(ADPribose)-polymerase-1
SHM: somatic hypermutation
