# Correction: The BRCT Domain of PARP-1 Is Required for Immunoglobulin Gene Conversion

**DOI:** 10.1371/journal.pbio.1002621

**Published:** 2018-03-01

**Authors:** Marcia N. Paddock, Ben D. Buelow, Shunichi Takeda, Andrew M. Scharenberg

The authors would like to clarify several issues recently raised by the PLOS Biology editors.

In the legend for Fig 1, it was not made clear that the E988K lane in panel C is the same as in Fig 6B. The authors have provided a corrected legend for Fig 1, which now states the origin of the E988K lane in panel C.

**Fig 1:**
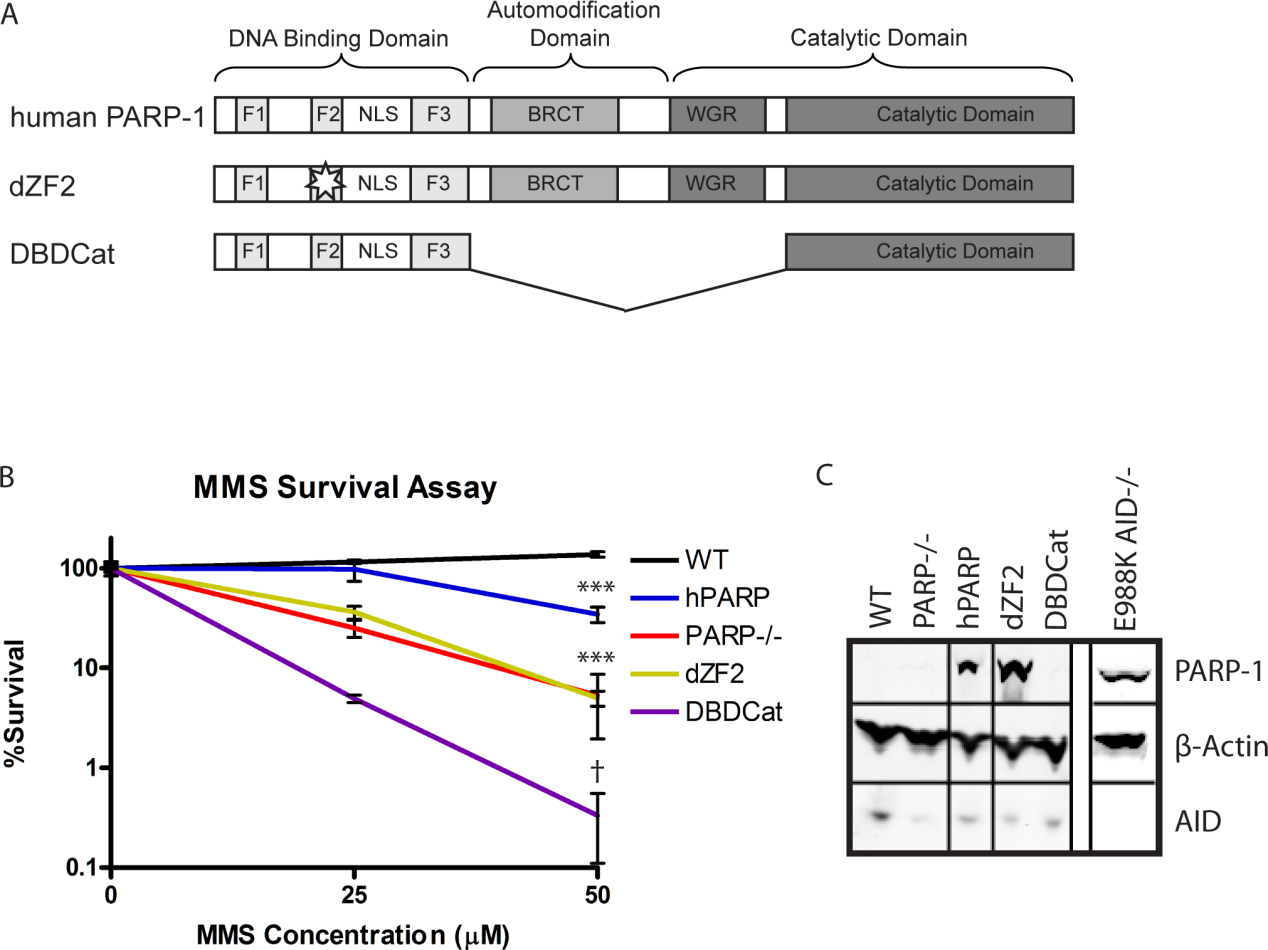
Functional effects of expression of human PARP-1 variants on survival in response to MMS-induced DNA damage. (A) Schematic of domains of human PARP-1 and variants. The functional domains of PARP-1 consist of a DNA binding domain (DBD), automodification domain (AMD), BRCT protein interaction domain (BRCT), and WGR/catalytic domain (WGR/Cat). The DBD contains 3 zinc finger domains, which are unusual in that they have specificity for DNA structure rather than sequence and recognize single strand breaks (SSBs) or double strand breaks (DSBs) [39,40]. The AMD contains the lysine residues that act as poly-ADP-ribose (PAR) acceptors [35]. The WGR/catalytic domain catalyzes PAR formation when the DBD is bound to DNA, and PARylation of the AMD is thought to serve as a signal to recruit DNA repair enzymes such as XRCC1 as well as facilitates the release of PARP-1 from the site of DNA damage [41]. The BRCT protein interaction domain is of unknown function, as it has been shown to be dispensable for PARP-1’s DNA repair functions in previous analyses [16]. hPARP: full length human PARP-1; dZF2: C125Y and C128Y mutations to prevent folding of the second zinc finger domain; DBDCat: DNA binding domain fused to a non-functional portion of the catalytic domain. (B) MMS survival assay comparing survival of the PARP-1 variants to MMS-induced DNA damage. Survival is measured by the ability to proliferate after 1 h of exposure to MMS at the indicated concentration. The experiment was performed in triplicate; error bars represent SEM. *** PARP-1^−/−^, dZF2, and hPARP p,.0001 compared to WT; PARP-1^−/−^ p,.0003 compared to hPARP; {p,.0001 compared to WT, p = .021 compared to PARP-1^−/−^; between PARP-1^−/−^ and dZF2 there is no significant difference. (C) Western blot showing levels of variant PARP-1 and AID expression with b actin as a loading control. The gel lane for the E988K mutant is taken from the original gel image shown in Figure 6B (lane labeled AID^−/−^), and is juxtaposed for comparison.

In the legend for Fig 2C, it was not made clear that these data are being re-used from Fig 1C. The authors have provided a corrected legend for Fig 2, which now states the origin of the data shown in Fig 2C.

**Fig 2:**
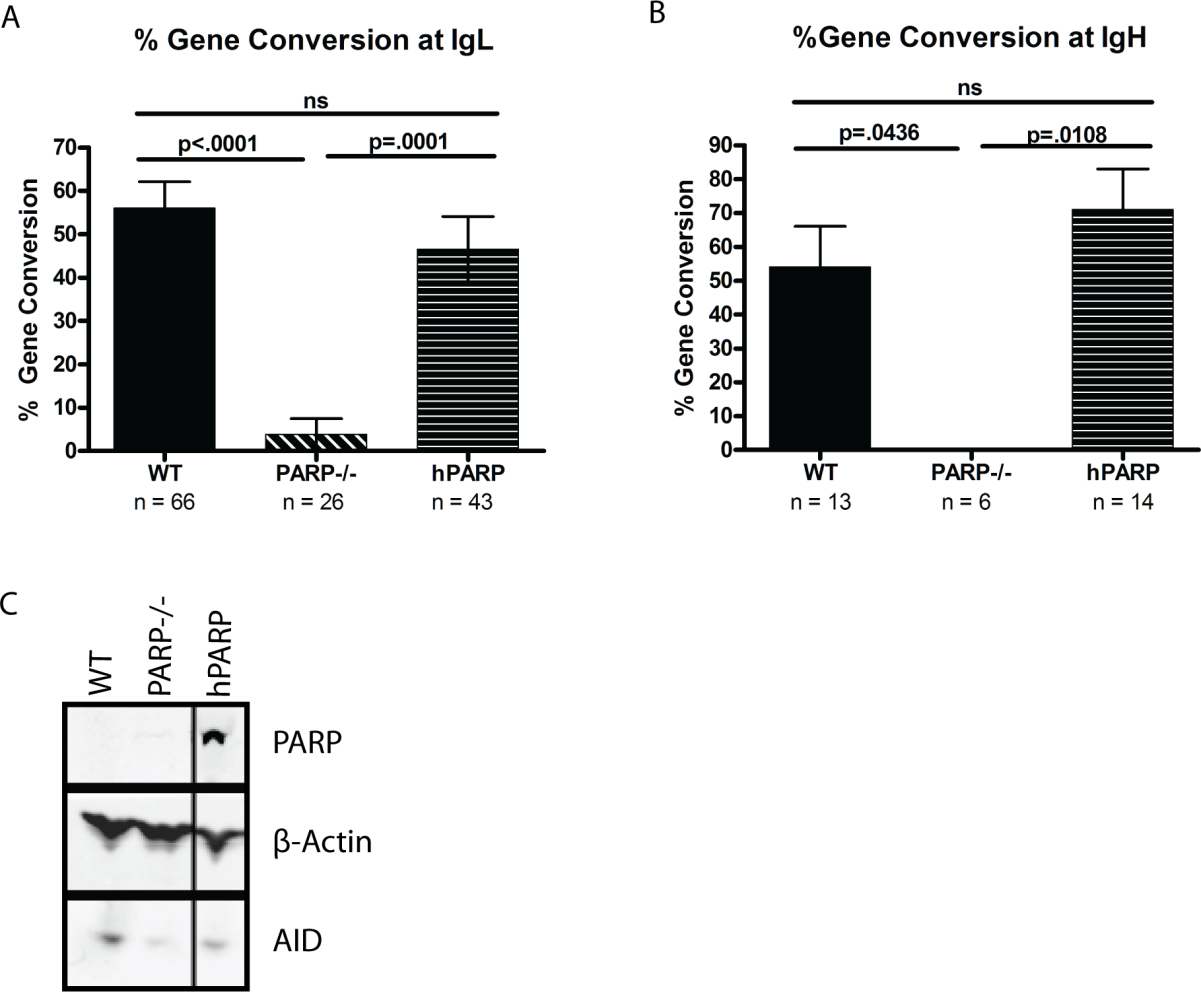
PARP-1 is required for Ig gene conversion. Gene conversion frequencies (+/− SEM) are indicated as a percentage of total mutations at IgL (A) and IgH (B). n = total number of mutations analyzed for each cell line at each locus. (C) Western blot showing PARP-1 and AID expression levels with b actin as a loading control reiterated from Fig 1C for convenience of viewing.

The published version of Fig 7C was constructed from original data shown in Figure 3B and 6B. The lanes 4–6 (noted as ggAID o/e) of Figure 3B are the source of lanes 1, 2, and 6 in the Fig 7C panel. The lanes 1–3 in Fig 6B are the source of the data for lanes 3, 4, and 5 in the Fig 7C panel. The composite panel was submitted without appropriate annotations in Fig 7C in error.

**Fig 7:**
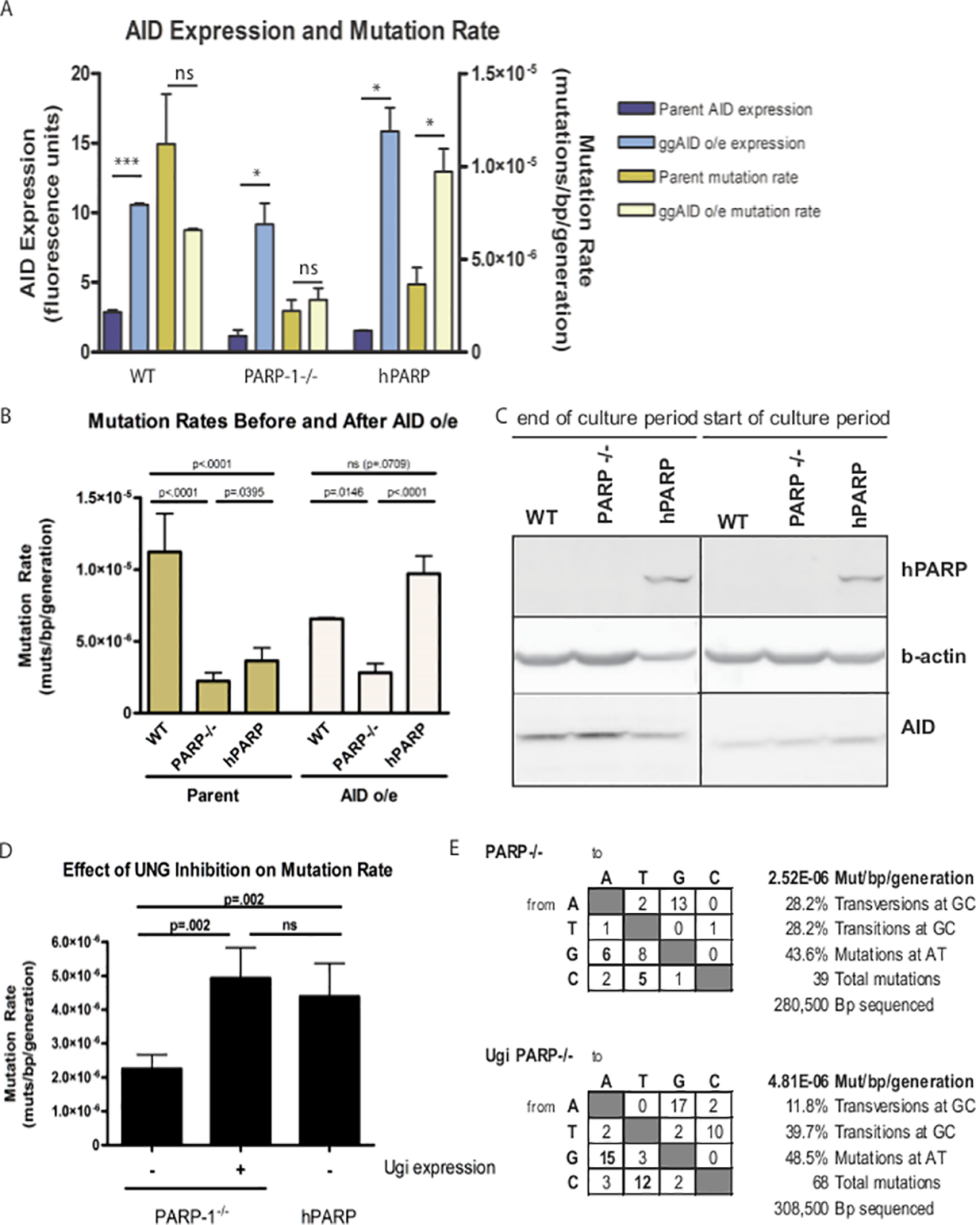
AID-induced lesions are more likely to undergo high-fidelity repair in PARP-1^−/−^ cells. (A) Mutation rate is limited in PARP-1^−/−^ cells, even when AID expression is restored. Blue bars show AID expression levels (mean +/− SEM) before (dark blue) and after (light blue) transduction with ggAID cDNA as measured by Western blot and quantified by LICOR Odessey infrared imaging, normalized to β actin. Yellow bars show total mutation rates (mean +/− SEM) before (dark yellow) and after (light yellow) transduction with ggAID cDNA, measured as total mutations/bp/generation for the indicated cell lines. *** *p* < .0001, * *p* < .05, *ns* = not significant. (B) Increased AID expression restores mutation rate in hPARP to WT levels, but PARP-1^−/−^ remain reduced. (C) Western blot showing PARP-1, AID, and β-actin levels at the beginning and the end of the culture period. (D) Mutation rate (mean +/− SEM when available) in PARP-1^−/−^ cells with and without UGI expression, compared to hPARP cells. (E) Tables showing the distribution of mutations in PARP-1^−/−^ and PARP-1^−/−^UGI cell lines. The changes are scored from the nucleotide indicated on the left to the nucleotide indicated on the top of the table.

As requested by the editors, the authors have re-run this experiment, using the same cell lines as used in the original experiments and, with this newly generated data, provided a corrected version of the figure here. The editors have verified the data underlying the corrected panel and are satisfied that these continue to uphold the conclusions from this figure. Note that the figure legend remains unchanged.

Methods:

Frozen aliquots of the cells used in the original experiments were expanded, lysed, and western blots for PARP, AID, and β-actin (as loading control) were generated. “Start of culture” cells were expanded for 4 days in culture, resulting in a total of c.a. 14 days expansion from a single clone. “End of culture” cells were expanded an additional 8 days for a total of c.a. 31 days total expansion. The resulting time differential approximately replicates the state of the cells analyzed in the original Fig 7C.

For each sample, protein content was quantitated, and Western blotting was performed using the methodology described in the paper. Secondary-antibody only blots were performed to assess for potential for non-specific bands related to the secondary antibody, and no bands were detected. Primary antibodies used for detection of specific proteins were 4C10-5 anti-PARP (bdbiosciences, catalog #556494), Anti-AICDA LS-C34861 (LS Biosciences), and anti-β–actin clone AC-74 (Sigma Aldrich). Western blot images were obtained on a Licor Odyssey Imaging System (Licor.com), and contrast-adjusted for illustrative purposes.

For compatibility with the original panel 7C legend, fragments corresponding to the “end of culture period” and “start of culture period” were excised from images of respective specific protein western blots in which all samples were processed and analyzed simultaneously.
