# Supplementary material for: The BRCT Domain of PARP-1 Is Required for Immunoglobulin Gene Conversion
Source: PLoS Biol. 2010 Jul 20;8(7):e1000428. doi: 10.1371/journal.pbio.1000428 (PMC2907289; doi:10.1371/journal.pbio.1000428)
Supplement: Figure S1 — Mutations observed in the IgL sequence of WT, PARP-1−/−, and hPARP cell lines. Point mutations are indicated below the reference sequence in red. Gene conversion events are indicated in boxes. The total number of sequences analyzed to generate these data were 93 WT, 184 PARP−/−, and 186 hPARP, all after 35 d in culture (70 generations). (0.24 MB PDF) [file pbio.1000428.s001.pdf]

WT   261 reads 35 day culture period

```
G C C G C C A A G T C C A A G A A A A A A C C C C C A C T A T C A C C A A A A A T C G A C A A A A T G T C A C A A T T T C A C G
                      T     T

                           [A A A A A A A]

A T G G G G G A A G A A A G A C C G A G A C G A G G T C A G C G A C T C A C C T A G G A C G G T C A G G G T T G T C C C G G C
                        T           G
                        T

C C C A A A T G C A G C A C C A C T G T T G T C T G C A C T C C C A C A G A A A T A G A C A G C C T C G T C A T C G G C T C G
                                                        T

                                           [C C T T C G T A G]
                                                   [T G]                                       [A]

G A C C C C A G T G A T G G T T A A T G T G T T T T G T G G A G C C G G A T A G G G A A C C G G A G A A T C G T G A A G G G A T
                                            C                   A                                         A
                                           [C C C G T G G A G C C G G A T G T G G A A C C G G A G A A T C G T G A A G G G A T]
                                           [G C]                                         [A]
                                           [G C]                         [G C]
                                           [G C]                         [C C A]
                                           [G C]
                                                                 [A G A T G T G G A A C C G G A G A A T C G T G A A G G G A T]

G T C C G A G G G T C T C T T G T C G T T G T C A T A G A T C A C A G T G A C A G G G G C A C T G C C A G G A G A C T T C T G

[G C C C G A G G G T C T C T C A T C A T C C C A G T A G A T C A C A G T G A C A A G G G C T C T G C C A G G A G A C T T C T G]
 [C]                   [G T T G G T]                         [G C A C]         [A]                         [T G C]
   [T]                 [G T T G G T G T T G T C A T A G A T C A G A G T G A C A G G G G C A C T G C C A G G T G C C T T C T G]
   [T T G A G G G T C T C T T G T C A T T C C A G]               [T]           [A]
                                                             [G]               [A]
[G T C C G A G G G T C T C T T G T C G T T G T C A T A G A T C A C A G T G A C A A]
                                                                               [A]

C T G G T G C C A G C C A T A A T A G T A A C T T C C A G C A T A G C T G C C A C C C C C G G A G C A G G T G A T C T T G A C
                    T               A                             C T                   T           G     A
                                                                  C
                                                                  C
[C T G G A A C C A G C C A T A G C T A T A G C T G C C C C C A G] C
       [A]                         [A T A A C T T C C A G G]                                                       [C]
[C T G G T A C C A G C C A T A G T A G C T C C T A T C C C C G G A G C A G G T G A T C T T G A C G G T T T C T C C C G G G T T]
       [A A]                                           [T A G]
       [A A]                                               [C G]         [T C A C T]
       [T A C C A G C C A T A A T A G T A A C T T C C A G C C G T]
     [A]
         [A]

G G T T T C T C C T G G G T T C G C T G A C A C C G A G G C C G G C T G A G T C A G C G C T G C C T G C A C C A G G G A A C C
T                                   A           T                                                         G

[T G C T G A C A C C G A G G A]


T G G A G A G G G A G A G G A G A G A G G G G A G A A A A C G G C A A T C A G T G A C G G C C C C G C
                                                          A     A                               T
```

PARP-/-   351 reads 35 day culture period

```
G C C G C C A A G T C C A A G A A A A A C C C C C A A A T C A C C A A A A A T C G A C A A A A T G T C A C A A T T T C A C G A
                T         G A                                                                                         G
                          G
                          G

T G G G G G A A G A A A G A C C G A G A C G A G G T C A G C G A C T C A C C T A G G A C G G T C A G G G T T G T C C C G G C C
                                  G                                                     A                           A

C C A A A T G C A G C A C C A C T G T T G T C T G T A T T C C C A C A G A A A T A G A C A G C C T C G T C A T C G G C T C G G

A C C C C A G T G A T G G T T A A T G T G T T T G T G G A G C C G G A T G G G G A A C C G G A G A A T C G T G A A G G G A T G
                                                        G               A               T

T C C G A G G G T C T C T T G T C G T T G T C A T A G A T C A C A G T G A C A G G G G C A C T G C C A G G A G A C T T C T G C
                    A         A                                                       T                                     T
                                                                            T
T G G T A C C A G C C A T A A T A G T A A C T T C C A G C A T A G C T G C C A C C C C C G G A G C A G G T G A T C T T G A C G
                                  A                 G                                 G

C C A C C C C C G G A G C A G G T G A T C T T G A C G G T T T C T C C T G G G T T T G C T G A C A C C G A G G C C G G C T G A
                                            C

G T C A G C G C T G C C T G C A C C A G G G A A C C T G G A G A G G G A G A G G A G A G A G G G G A G A A A A C G G C A A T C
          T

A G T G A C G G C C C C G C
                T
```

<u>hPARP</u>   368 reads 35 day culture period

```
G C C G C C A A G T C C A A G A A A A A C C C C C A A A T C A C C A A A A A T C G A C A A A A T G T C A C A A T T T C A C G A
                A         G T
                          G T
                          G T

T G G G G A A G A A A G A C C G A G A C G A G G T C A G C G A C T C A C C T A G G A C G G T C A G G G T T G T C C C G G C C
                                  G

C C A A A T G C A G C A C C A C T G T T G T C T G T A T T C C C A C A G A A A T A G A C A G C C T C G T C A T C G G C T C G G
                                                  C                             G

                                                A T G C
                                                T C A T T C C C A C A G T
                                                  C T A
                                                A T G C                 T
A C C C C A G T G A T G G T T A A T G T G T T T G T G G A G C C G G A T A G G G A A C C G G A G A A T C G T G A A G G G A T G
                                          C T T G T G G A G C C G G A T G T G G A A C C G G A G A A T C G T G A A G G G A T G
                                          G C
                                          G C T G T G G A G C C G G A T G C G G A A C C G G A G A A T C G T C A A G G G A T G
                                          G C C G T G G A G C C G A A T T T
                                          C C C
T C C G A G G G T C T C T T G T C G T T G T C A T A G A T C A C A G T G A C A G G G G C A C T G C C A G G A G A C T T C T G C
                                                              G C

T T
  T T G A G G G T C T C T T G T C G T G G T C A T A G A T C A C A G T G A C A G G G G C A C T G C C A G G A G A C T T C T G C
T C C G A G G G T C T C T T G T C G T T G C T A T A G A T C A G                                             T G C C T T C T G C
  T C G A G G G T C T G T T G G T G T T G T C A T A G A T C A C A G T G A C A G G G G C A C T G C C A G G T G C C T T C T G C
  T
T G G A A C C A G C C A T A A T A A T A A C T T C C A G C A T A G C T G C C A C C C C C G G A G C A G G T G A T C T T G A C G
                                                      A           G

                                  G
T G G A A C C A G C C A T A A T A A T A A C T T C C A C
T G G T A C C A G C C A T A A T A G
T G G T A C C A G C C A T A A T A G
                                  G
                                  G
C C A C C C C C G G A G C A G G T G A T C T T G A C G C C A C C C C C G G A G C A G G T G A T C T T G A C G G T T T C T C C T
                                                                                                                  C C T C C C

G G G T T T G C T G A C A C C G A G G C C G G C T G C T G A G T C A G C G C T G C C T G C A C C A G G G A A C C T G G A G A G
                      A                                                                                                   G
A
A
G G A G A G G A G A G A G G G G A G A A A A C G G C A A T C A G T G A C G G C C C C G C
                                            A                         A
```
