# Supplementary figures and images for: The BRCT Domain of PARP-1 Is Required for Immunoglobulin Gene Conversion
Source: PLoS Biol. 2010 Jul 20;8(7):e1000428. doi: 10.1371/journal.pbio.1000428 (PMC2907289; doi:10.1371/journal.pbio.1000428)

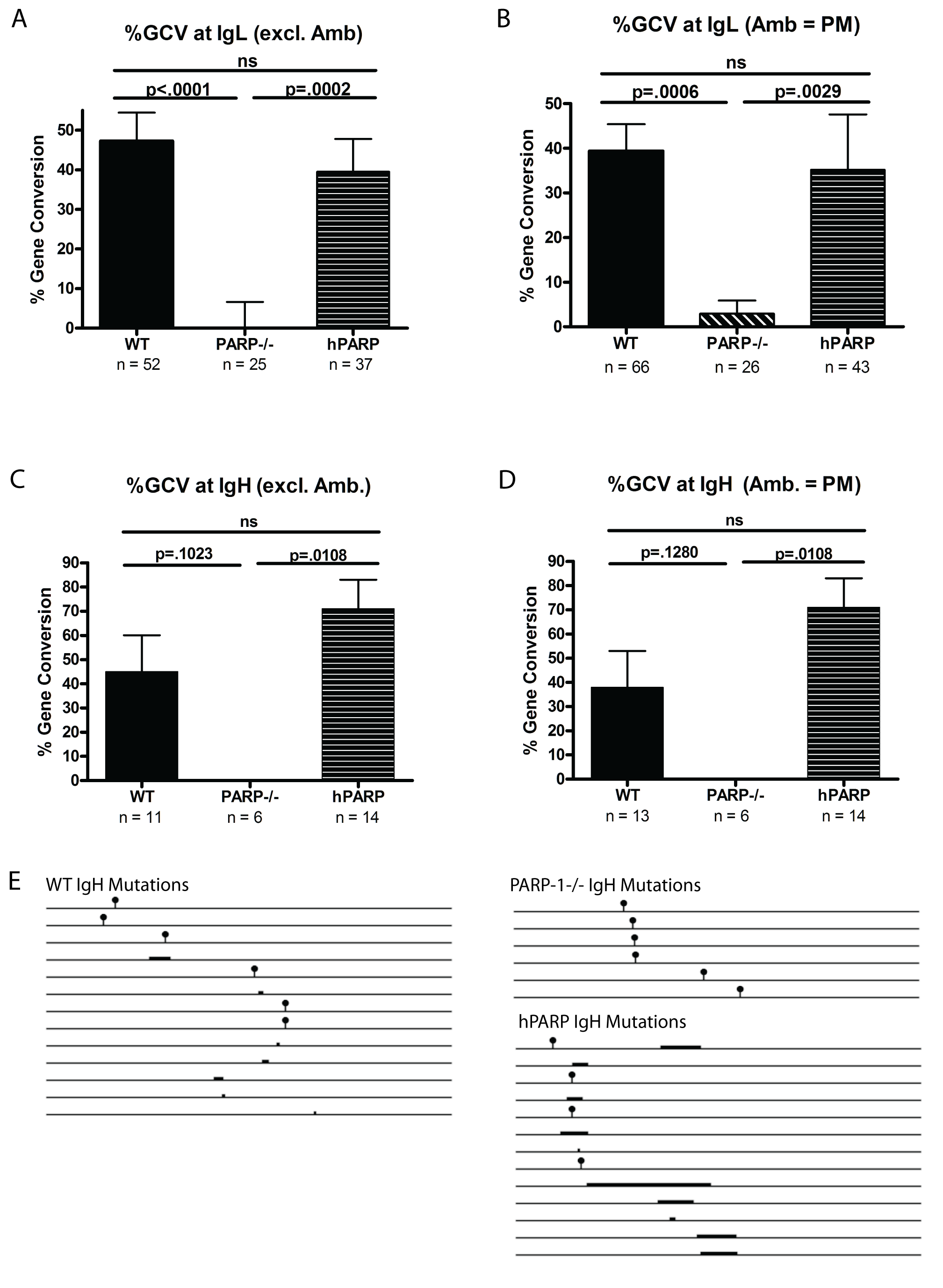

Supplement: Figure S2 — Different assessments of GCV do not change the dependence on PARP-1. Gene conversion frequencies at IgL when (A) percent gene conversion is calculated when “ambiguous” mutations (those that match upstream pseudogenes, but occur in isolation) are excluded from analysis or (B) when “ambiguous” mutations are categorized as point mutations. (C) and (D) are the same analyses at the IgH locus. (E) Line drawing depicting point mutations (lollipops) and gene conversion events (bars) in WT, PARP-1−/−, and hPARP at the IgH locus. (0.75 MB TIF) [file pbio.1000428.s002.tif]

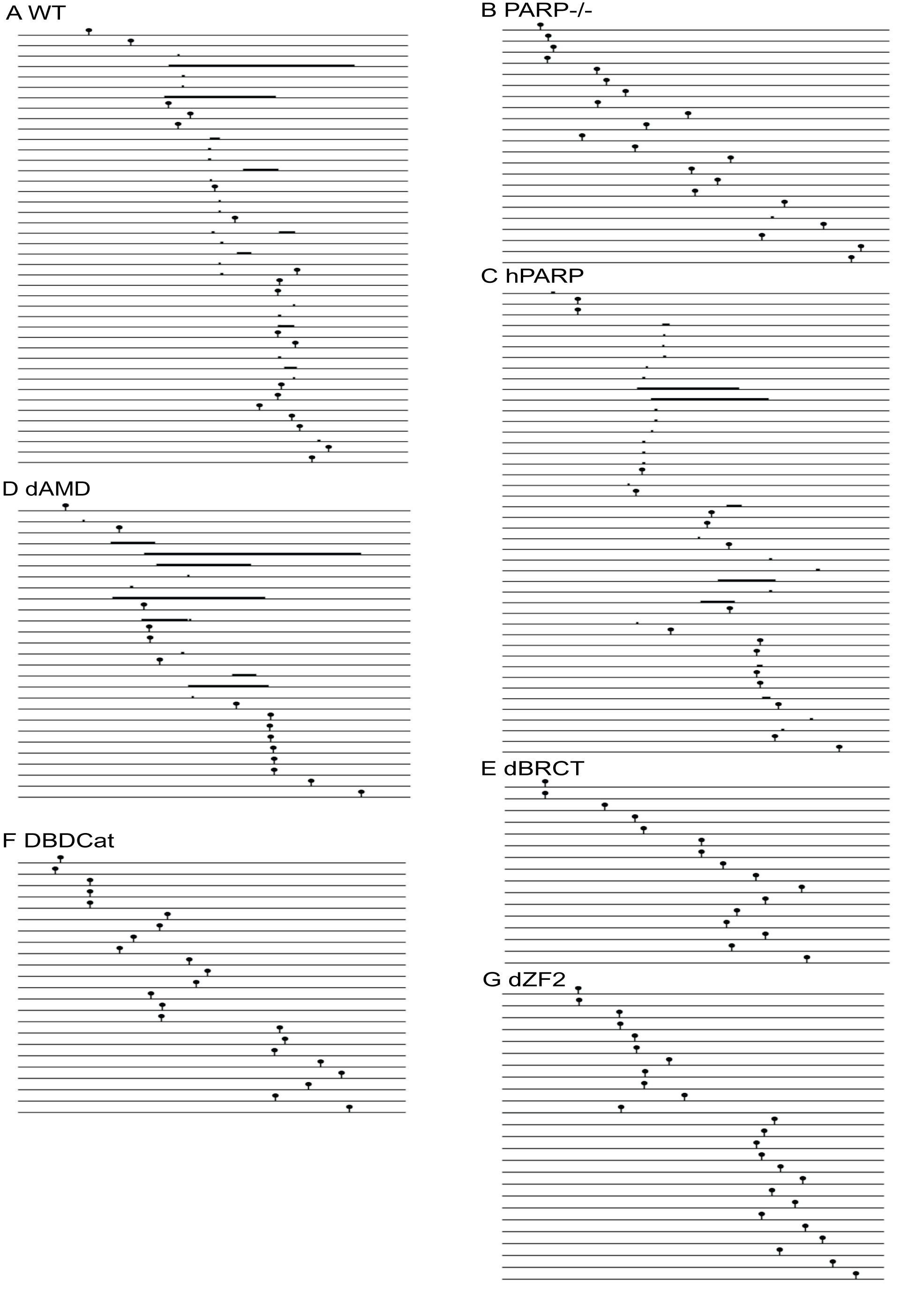

Supplement: Figure S3 — Schematic representation of mutations observed at IgL in multiple PARP-1 variants. Line drawing depicting representative point mutations (lollipops) and gene conversion events (bars) at IgL in each of the cell lines used in this study after 30 d in culture (60 generations). (A) WT, 58 total mutations in 168 reads. (B) PARP-1−/−, 22 total mutations in 175 reads. (C) hPARP, 53 total mutations in 106 reads. (D) dAMD, 27 total mutations in 79 reads. (E) dBRCT, 16 total mutations in 236 reads. (F) DBDCat, 23 total mutations in 255 reads. (G) dZF2, 26 total mutations in 430 reads. (0.56 MB TIF) [file pbio.1000428.s003.tif]
