# Supplementary material for: The BRCT Domain of PARP-1 Is Required for Immunoglobulin Gene Conversion
Source: PLoS Biol. 2010 Jul 20;8(7):e1000428. doi: 10.1371/journal.pbio.1000428 (PMC2907289; doi:10.1371/journal.pbio.1000428)
Supplement: Figure S4 — Background mutation rates of polymerase and DT40s in culture. (A) Mutations accumulated in an irrelevant gene in DT40 cells over 8 wk (120 generations) in culture when amplified with Pfx (Invitrogen). (B) Mutations accumulated in the constant region of IgL in DT40 cells over 6 wk (84 generations) in culture when amplified with Pfx (Invitrogen). (C) Comparison of mutations accumulated in the variable region of IgL in AID−/− PARPWT DT40 cells over 10 wk (140 generations) in culture when amplified with Pfx (Invitrogen) or Pfu (Stratagene). (D) Comparison of mutations accumulated in the variable region of IgL in Ugi expressing PARP-1−/− DT40 cells over 3 wk (46 generations) in culture when amplified with Pfx (Invitrogen) or Pfu (Stratagene). (0.02 MB PDF) [file pbio.1000428.s004.pdf]

A

Irrelevant Gene

Pfx

from \ to	a	t	g	c
a		0	0	0
t	0		0	1
g	0	0		1
c	0	2	0	

	**2.50E-07**	**Mut/bp/generation**
	25.0%	Transversions at GC
	50.0%	Transitions at GC
	25.0%	Mutations at AT
	4	Total mutations
4	133200	Bp sequenced

B

Constant Region

Pfx

from \ to	**A**	**T**	**G**	**C**
**A**		2	0	1
**T**	0		0	2
**G**	1	0		2
**C**	0	4	0	

**7.88E-07**	**Mut/bp/generation**
16.7%	Transversions at GC
41.7%	Transitions at GC
41.7%	Mutations at AT
12	Total mutations
181,200	Bp sequenced

C

AID-/- IgL-V

Pfx

from \ to	**A**	**T**	**G**	**C**
**A**		1	1	0
**T**	0		0	2
**G**	3	0		1
**C**	3	1	0	

**8.17E-07**	**Mut/bp/generation**
33.3%	Transversions at GC
33.3%	Transitions at GC
33.3%	Mutations at AT
12	Total mutations
102,000	Bp sequenced

AID-/- IgL-V

Pfu

from \ to	**A**	**T**	**G**	**C**
**A**		0	0	0
**T**	0		0	0
**G**	3	0		0
**C**	2	3	0	

**6.14E-07**	**Mut/bp/generation**
25.0%	Transversions at GC
75.0%	Transitions at GC
0.0%	Mutations at AT
8	Total mutations
93,000	Bp sequenced

D

PARP-1-/- Ugi

Pfx

from \ to	a	t	g	c
a		0	2	0
t	0		0	0
g	4	0		0
c	1	0	0	

**4.91E-06**	**Mut/bp/generation**
14.3%	Transversions at GC
57.1%	Transitions at GC
28.6%	Mutations at AT
7	Total mutations
31000	Bp sequenced

PARP-1-/- Ugi

Pfu

from \ to	a	t	g	c
a		1	0	0
t	1		0	1
g	5	0		0
c	1	1	0	

**4.14E-06**	**Mut/bp/generation**
10.0%	Transversions at GC
60.0%	Transitions at GC
30.0%	Mutations at AT
10	Total mutations
52500	Bp sequenced
